# *Mycn* Reactivates the Cell Cycle in Adult Cardiomyocytes and Promotes Cardioprotection in Myocardial Infarction

**DOI:** 10.1161/JAHA.125.046146

**Published:** 2026-04-09

**Authors:** Aina Hirofuji, Hiroki Tanaka, Yuki Tsujita, Ryo Okubo, Ryohei Ushioda, Yumiko Fujii, Yusuke Ono, Michihiro Hashimoto, Megumi Kanao‐Kanda, Yusuke Mizukami, Hiroyuki Kamiya, Kyohei Oyama

**Affiliations:** ^1^ Department of Cardiac Surgery Asahikawa Medical University Asahikawa Japan; ^2^ Laboratory of Molecular Diagnostics and Therapeutics, Center for Intractable Diseases and ImmunoGenomics National Institutes of Biomedical Innovation Osaka Japan; ^3^ Division of Tumor Pathology, Department of Pathology Asahikawa Medical University Hokkaido Japan; ^4^ Institute of Biomedical Research Sapporo‐Higashi Tokushukai Hospital Sapporo Japan; ^5^ Department of Advanced Medical Science Asahikawa Medical University Asahikawa Japan; ^6^ Department of Anesthesiology Wakayama Medical University School of Medicine Wakayama Japan; ^7^ Department of Anesthesiology & Critical Care Medicine Asahikawa Medical University Asahikawa Japan; ^8^ Division of Gastroenterology, Department of Medicine Asahikawa Medical University Asahikawa Japan

**Keywords:** cardiomyocyte, cardioprotection, cell cycle re‐entry, *Mycn*, myocardial infarction, Basic Science Research

## Abstract

**Background:**

Adult cardiomyocytes are terminally differentiated with limited capacity for cell cycle re‐entry. However, recent studies have shown that cycling cardiomyocytes may exert cardioprotective effects after myocardial infarction (MI). The *Myc* family—*Myc*, *Mycl*, and *Mycn*—regulates cell cycle progression and plasticity, raising the possibility that specific isoforms could reactivate cardiomyocyte cycling and therefore enhance cardiac repair in MI. This study evaluated the effects of *Myc* isoforms on cardiomyocyte cell cycle activation and cardiac outcomes after MI.

**Methods:**

Cardiomyocyte‐specific overexpression of *Myc*, *Mycl*, or *Mycn* was induced in adult mice using adeno‐associated virus 9 vectors driven by the cardiac troponin T promoter. Cell cycle activity, remodeling, and function were assessed by RNA sequencing, immunohistochemistry, and echocardiography. Cardioprotection was evaluated in MI models.

**Results:**

*Mycn* elicited the most robust cell cycle gene expression among the *Myc* isoforms. *Mycn* overexpression markedly enhanced cardiomyocyte re‐entry—evidenced by increased 5‐bromo‐2′‐deoxyuridine incorporation and histone H3 phosphorylation—and induced hypertrophic growth. Transcriptomic profiling revealed *Mycn*‐specific upregulation of extracellular matrix and paracrine signaling genes, which are typically enriched in neonatal cardiomyocytes and also linked to cardioprotection. In MI models, *Mycn* preserved cardiac contractility, reduced infarct size, and increased capillary density in peri‐infarct regions.

**Conclusions:**

*Mycn* exerts robust biological effects in the adult heart, including reactivation of the cardiomyocyte cell cycle and promotion of cardioprotection following ischemic injury. Its effects likely involve induction of a neonatal‐like transcriptional program that fosters a stress‐adaptive microenvironment.

**Nonstandard Abbreviations and Acronyms**
AAV: adeno‐associated virus
BrdU: 5‐bromo‐2′‐deoxyuridine
cTnT: cardiac troponin T
GO: Gene Ontology
pH3: phospho‐histone H3
RNA‐seq: RNA sequencing
WGA: wheat germ agglutinin

**Clinical Perspective**

**What Is New?**

- Among the *Myc* family genes (*Myc*, *Mycl*, *Mycn*), *Mycn* induces cardiomyocyte cell cycle activation in the adult heart under identical experimental conditions.
- Cardiac‐specific *Mycn* expression confers cardioprotection in myocardial infarction.

**What Are the Clinical Implications?**

- *Mycn* and *Mycn*‐activated pathways may represent therapeutic targets to enhance cardiomyocyte stress tolerance during ischemic injury.

Cardiomyocytes in adult mammals, including humans, exhibit an extremely limited capacity for cell division, rendering the heart one of the least regenerative organs in the body.^1^, ^2^ Following myocardial injury—such as myocardial infarction (MI) or myocarditis—irreversible fibrosis typically ensues, leading to progressive and permanent cardiac dysfunction. In contrast, neonatal mammals retain an intrinsic capacity for cardiomyocyte proliferation during a narrow postnatal window—within 1 week of birth in mice—which enables them to regenerate heart tissue.^3^, ^4^ This observation has spurred investigations into the molecular mechanisms governing cardiomyocyte cell cycle, with the aim of developing new strategies to support cardiac repair or protection.

Postnatal mammalian cardiomyocytes are widely considered terminally differentiated and rarely reenter the cell cycle. However, accumulating evidence suggests that a small subset of cardiomyocytes retains the ability to reengage with the cell cycle at a very low frequency—~0.1% per year in both humans and mice.^5^, ^6^ This activity appears to be enhanced under pathological conditions; for example, in surgically induced MI model mice, cardiomyocytes in infarct border zones exhibit a marked increase in cell cycle re‐entry.^7^, ^8^ Notably, genetic ablation of endogenously cycling cardiomyocytes significantly worsens cardiac function following MI in mice.^9^ Although the mechanism remains to be fully elucidated, these findings suggest cardiomyocyte cell cycle activation confers cardiac protection in hearts under ischemic stress.

The *Myc* family of transcription factors—comprising *Myc*, *Mycl*, and *Mycn*—are well‐established regulators of cellular proliferation, metabolism, and microenvironmental remodeling. Each member exhibits tissue‐ and context‐specific functions^10^, ^11^; for example, *Mycl* is critical for pancreatic cell division,^12^ whereas *Mycn* plays a critical role in cardiomyocyte proliferation during embryonic heart development.^13^, ^14^ Given these roles, the promotion of cell cycle activity is considered a core function of the *Myc* gene family. However, previous studies have shown that forced expression of *Myc* alone in adult cardiomyocytes induces only partial cell cycle re‐entry and fails to drive full proliferation.^15^, ^16^ In contrast, the functional roles of *Mycl* and *Mycn* in mature cardiomyocytes remain largely uncharacterized, raising the possibility that they may differ in their capacity to activate the cardiomyocyte cell cycle.

We hypothesized that individual *Myc* isoforms possess distinct functional capacities in adult cardiomyocytes, with specific isoforms capable of promoting more cell cycle activation and providing protection in the setting of MI. To test this hypothesis, we induced cardiomyocyte‐specific overexpression of *Myc*, *Mycl*, and *Mycn* in adult mice and compared their effects on cell cycle activation. Additionally, we assessed whether any *Myc* family member conferred functional cardioprotection in the context of MI.

## METHODS

### Data Availability

RNA sequencing (RNA‐seq) data were deposited in the DDBJ BioProject database under the accession number PRJDB35457. The data, protocols, and study materials that support the findings of this study are available from the corresponding author upon request.

### Construction of Adeno‐Associated Virus Transfer Vectors

A cTnT (cardiac troponin T) promoter‐driven *GFP*‐expressing adeno‐associated virus (AAV) transfer plasmid (pENN.AAV.cTNT.PI.eGFP.WPRE.rBG; Addgene #105543) was used as the backbone. The plasmid was digested with *BmtI* and *KpnI* to replace the *GFP* coding sequence with *GFP*‐V5, *Myc*‐V5, *Mycl*‐V5, or *Mycn*‐V5 using the In‐Fusion HD Cloning Kit (Clontech, 639 649). The sequences of the inserted constructs are provided in Data S1. The resulting AAV transfer plasmids (pAAV‐cTnT‐*GFP*‐V5, pAAV‐cTnT‐*Myc*‐V5, pAAV‐cTnT‐*Mycl*‐V5, and pAAV‐cTnT‐*Mycn*‐V5) were packaged into AAV9 particles following a protocol described by Kimura and colleagues,^17^ or using the AAV production service provided by VectorBuilder.

### Animal Studies

All mice used in this study were housed under specific pathogen‐free conditions at the Asahikawa Medical University. All animal experiments were approved by the Institutional Animal Care and Use Committee of the Asahikawa Medical University. Only male mice were used to minimize biological variability and maintain focus on the primary objective of evaluating *Myc* family transgene effects in cardiomyocytes.

### Mice and AAV Vector Administration

Male C57BL/6 mice were purchased from Japan SLC, Inc. At 6 weeks of age, AAV vectors (1.5×10^11^ viral genome copies in 100 μL per mouse) were administered via tail vein injection. Starting on the day of AAV vector administration, mice were provided with drinking water containing 5‐bromo‐2′‐deoxyuridine (BrdU, 0.4 mg/mL) continuously for 2 weeks for end–point assay of the number of cardiomyocytes that had reentered the S‐phase. At the end of the treatment period, cardiac function was assessed by transthoracic echocardiography (Venu Go, GE HealthCare), and hearts were harvested for downstream analyses.

### MI Model Mice: Permanent Left Anterior Descending Artery Ligation

Male C57BL/6 mice at 6 weeks of age were randomly allocated to the control (GFP) group and *Mycn* groups and then randomly assigned to either sham or MI surgery. The mice were initially anesthetized in an induction chamber with isoflurane (2% volume, air 500 mL/min) and subcutaneously injected with buprenorphine (0.05 μg/g). The mice were placed on a sterile heating plate (ThermoStar, Intellibio) set at 40 °C in the supine position with a face mask connected to an anesthesia system (MK‐V100, Muromachi). Isoflurane was adjusted to provide a maintenance level (1.4%–2.4% volume, air 500 mL/min) throughout the procedure. Anesthesia was monitored by observing the depth and rate of respiration, mucous membrane color, reflexes and tail pinch, and the overall appearance of muscle relaxation.

All surgical procedures were performed using a surgical loupe (Orascoptic) and a sterile technique. The surgical sites (neck and chest wall) were shaved and prepped with 10% iodine. A small midline cervical incision of 2 mm was made, and the skin, muscle, and adipose tissue covering the trachea were gently separated with blunt surgical instruments to expose the thoracic cavity using blunt forceps until the left lung and pericardium were exposed. The retractors were repositioned onto the upper and lower ribs of the intercostal space to secure the surgical field of the heart.

For sham surgery mice, the thoracic cavity was kept open for 10 seconds, without further manipulation within the thoracic cavity, and the chest wall and skin were sutured closed. In MI model mice, the pericardium was bluntly dissected to expose the left anterior descending artery (LAD). The LAD was then ligated as centrally as possible using a 7‐0 prolene suture (Ethicon). LAD occlusion was verified by the blanching of the left ventricular muscle downstream of the ligation. The retractors were removed, and as much air as possible was pushed out of the thoracic cavity before the chest wall and skin were sutured with a 5‐0 prolene suture (Ethicon). Mice received postoperative oral analgesia via meloxicam (Sawai) in drinking water (0.02 mg/mL, for ~5 mg/kg per day) for 5 days. The mortality rate associated with surgeries was ~11% for sham mice and 37% for MI model mice. LAD ligation was performed at a distal site to reduce perioperative mortality. In this MI model, cardiac function remained relatively preserved for 2 weeks post–infarction and declined significantly by 4 weeks.

### Transthoracic Echocardiography on Mice

Anesthesia maintenance and monitoring were performed in the same manner as during the surgical procedures. Mice were initially anesthetized in an induction chamber with isoflurane (2% volume, air 500 mL/min), and on a heating plate (ThermoStar, Intellibio) set at 40 °C, gently restrained in the supine position, with a face mask delivering isoflurane anesthesia (1.4%–2.4% volume, air 500 mL/min) throughout the echocardiography.

All echocardiography was performed with the L10‐22 linear transducer probe and Venu Go echocardiography system (GE HealthCare), which included an integrated limb lead ECG. The ECG electrodes were secured topically on the limbs, the fur was gently removed with hair removal cream, and ultrasound contact gel was applied to the transducer probe. Initial B‐mode images were acquired in the parasternal long‐axis view, ensuring clear visualization of both the apex and aortic valves. Subsequently, B‐mode short‐axis images were obtained by rotating the ultrasound transducer ~90°, enabling the identification of the mitral valve papillary muscles, followed by M‐mode images in the same visual field. Left ventricular dimensions were calculated from parasternal long‐axis images using the Venu Go software.

### Cardiomyocyte Isolation

Cardiomyocytes were isolated using Langendorff perfusion digestion, as previously described, with minor modifications.^18^ Briefly, the hearts were washed via coronary perfusion with calcium‐free Tyrode's buffer (126 mM NaCl, 5.4 mM KCl, 0.33 mM NaH_2_PO_4_, 1 mM MgCl_2_, 10 mM HEPES, 10 mM glucose, 20 mM taurine, pH 7.4) supplemented with 20 mM 2,3‐butanedione monoxime (BDM; Cayman Chemical, 20828) for 2 minutes. Enzymatic digestion was then performed at 37 °C by perfusing with 0.69 U/mL Liberase TH (Roche, 5401151001) prepared in calcium‐free Tyrode's buffer containing 20 mM BDM for 10 to 12 minutes. The digested hearts were mechanically dissociated in ice‐cold KB solution (20 mM KCl, 10 mM KH_2_PO_4_, 70 mM potassium glutamate, 1 mM MgCl_2_, 25 mM glucose, 20 mM taurine, 0.5 mM EGTA, 10 mM HEPES, 0.1% albumin, pH 7.4). The resulting cardiomyocyte suspensions were passed through a 100 to 200 mm cell strainer to remove tissue debris and purified by low‐speed centrifugation (100× ***g*** for 1 minute), repeated 3 times. This procedure yielded cardiomyocytes with an approximate purity of 90%.

### Reverse Transcription Quantitative Polymerase Chain Reaction and RNA‐Seq

Total RNA was extracted from purified cardiac myocytes using TRIzol reagent (Thermo Fisher Scientific, 15596026) and further purified using the NucleoSpin RNA kit (MACHEREY‐NAGEL, 740955.250). cDNA was synthesized from purified RNA using ReverTra Ace qPCR RT Master Mix (TOYOBO, FSQ‐201). Quantitative polymerase chain reaction (qPCR) was performed using the THUNDERBIRD Next SYBR qPCR Mix (TOYOBO, QPX‐201X5), according to the manufacturer's instructions. The gene‐specific primers used in this study are listed in Data S2.

RNA sequencing was performed by Rhelixa Inc. (Tokyo, Japan) using ribosomal RNA depletion and strand‐specific library preparation methods. Paired‐end sequencing (150 bp × 2) was performed, generating ~40 million reads for each sample. Each group included 2 biological replicates each. RNA‐seq data were deposited in the DDBJ BioProject database under the accession number PRJDB35457.

### RNA‐Seq Data Analysis

Raw sequencing reads were preprocessed using fastp (v0.23.2) with default settings.^19^ Transcript abundance was quantified using Salmon (v1.9.0) with the ‐‐gcBias option enabled.^20^ The reference transcriptome and corresponding general transfer format annotation files were obtained from GENCODE Release M25 (GRCm38.p6). Transcript‐level abundances were summarized to the gene level using the tximport package (v1.30.0), and differential gene expression analysis was performed using DESeq2.^21^ Differentially expressed genes were defined as those with a false discovery rate <0.05 and fold change ≥2.

A list of differentially expressed gene was subjected to Gene Ontology (GO) enrichment analysis using the Gene Ontology Consortium's web‐based enrichment tool (https://geneontology. org/). GO terms in the Biological Process category with false discovery rates <0.05 were considered significantly enriched. To reduce redundancy, enriched GO terms were further processed using Reduce and Visualise Gene Ontology (http://revigo.irb.hr) with the default semantic similarity measure and a medium similarity cutoff (0.7). GO terms with a dispensability score <0.05 were retained as representative enriched terms.

### Histopathological Analysis

For histopathological analysis, formalin‐fixed paraffin‐embedded tissue specimens were prepared. For bright‐field microscopy, sections were stained using standard protocols for hematoxylin and eosin and picrosirius red. For immunohistochemical analysis, sections underwent sequential treatment including deparaffinization, rehydration, quenching of endogenous peroxidase activity, and antigen retrieval. Sections were then incubated with the following primary antibodies: anti‐V5 (Sigma‐Aldrich, V8012), anti‐BrdU (ThermoFisher Scientific, B35128), and anti‐phospho‐histone H3 (pH3; Abcam, ab5176). Detection was performed using horseradish peroxidase‐conjugated antimouse and antirabbit secondary antibodies (Vector Laboratories). For immunofluorescent staining, sections were incubated with the following primary antibodies: anti‐HSP47 (Abcam, ab109117) and anti‐Ki67 (ThermoFisher Scientific, 740008T). Detection was performed using an antirabbit secondary antibody conjugated with Alexa Fluor 555 (ThermoFisher Scientific, A‐31572) and an antirat secondary antibody conjugated with Alexa Fluor 647 (Abcam, ab150155). Cell membranes were stained using wheat germ agglutinin conjugated with Alexa Fluor 488, and Hoechst 33342 (FUJIFILM, 346‐07951) was used for nuclear counterstaining. Fluorescent and bright‐field images were obtained using a BZ‐X800 fluorescence microscope (Keyence Co.) and a NanoZoomer S60 virtual slide scanner (Hamamatsu Photonics Co.), respectively.

Mouse heart tissues obtained from MI and sham surgeries were fixed and transversely sectioned into 2‐mm slices. Among these, the slice exhibiting the largest infarct area was selected for quantitative analysis. Virtual slides stained with picrosirius red, as well as immunohistochemistry for BrdU and pH3, were analyzed using QuPath software to quantify infarct area and the percentage of positively stained nuclei.

### Statistical Analysis

Statistical analyses were performed using GraphPad Prism software. For multiple comparisons, 1‐way ANOVA followed by Tukey's post hoc test was performed. In addition, permutation‐based ANOVA was conducted as a complementary nonparametric approach, and the corresponding results are provided in Table S1. Unpaired 2‐tailed Student's *t* tests were used for 2‐group comparisons. Statistical significance was set at *p* < 0.05. Exact adjusted *P* values for all pairwise comparisons are provided in Table S2. Data visualization was performed using GraphPad Prism software and the ggplot2 package in R. Data are represented with biological replicates as individual points and bars as mean±SD.

## RESULTS

### AAV‐Induced Overexpression of *Myc* Isoforms in Adult Mouse Cardiomyocytes

**Figure 1:**
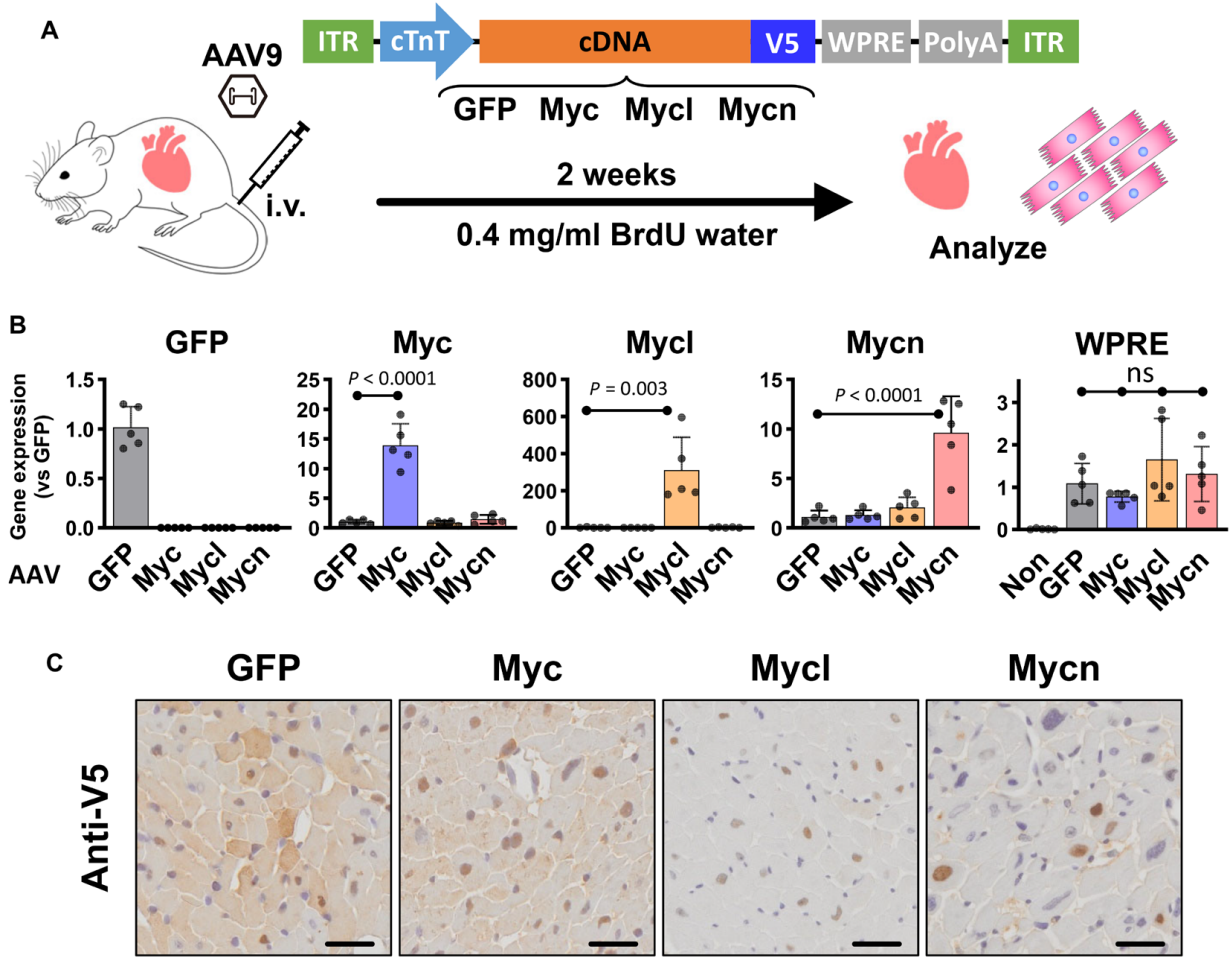
Cardiomyocyte‐specific *Myc* family gene transduction model. **A**, Experimental design: adeno‐associated virus vectors encoding *GFP*, *Myc*, *Mycl*, or *Mycn* were administered intravenously. Hearts or isolated cardiomyocytes were harvested 2 weeks post–injection for analysis. 5‐bromo‐2′‐deoxyuridine was administered via drinking water during the 2‐week period for histological assessment of DNA synthesis. **B**, Validation of transgene expression: cardiomyocytes were isolated, and real‐time reverse transcription‐polymerase chain reaction was used to confirm expression of *Myc* family genes. Statistical analyses were performed using 1‐way ANOVA followed by Tukey's post hoc test. **C**, Validation of cardiomyocyte‐specific expression: Immunostaining confirmed cardiomyocyte‐specific expression of the transgenes. Scale bar: 25 μm. AAV indicates adeno‐associated virus; BrdU, 5‐bromo‐2′‐deoxyuridine; cTnT, cardiac troponin T; ITR, inverted terminal repeats; ns, not significant; and WPRE, woodchuck hepatitis virus posttranscriptional regulatory element.

To investigate the effects of *Myc* family factors on the cell cycle in adult murine cardiomyocytes, an AAV9 gene delivery system was used. AAV9 vectors encoding cardiac‐specific *Myc*, *Mycl*, *Mycn*, and a *GFP* control were intravenously injected into 6‐week‐old mice, and the hearts were harvested for analysis 2 weeks post–injection (Figure 1A). RNA was extracted from purified cardiomyocytes, and the expression of *Myc*, *Mycl*, *Mycn*, and *GFP* was measured using real‐time reverse transcription‐PCR, confirming the overexpression of the *Myc* family factors (Figure 1B). Using a common woodchuck hepatitis virus posttranscriptional regulatory element primer set, overall transgene expression was confirmed to be comparable across groups. Immunohistochemistry with a V5 tag‐specific antibody demonstrated that each *Myc* family factor was specifically expressed in cardiomyocytes within the myocardial tissue (Figure 1C). These data confirmed the achievement of cardiac‐specific expression of all *Myc* family members.

### The Effects of *Myc* Family Factors on the Transcriptome in Cardiomyocytes

**Figure 2:**
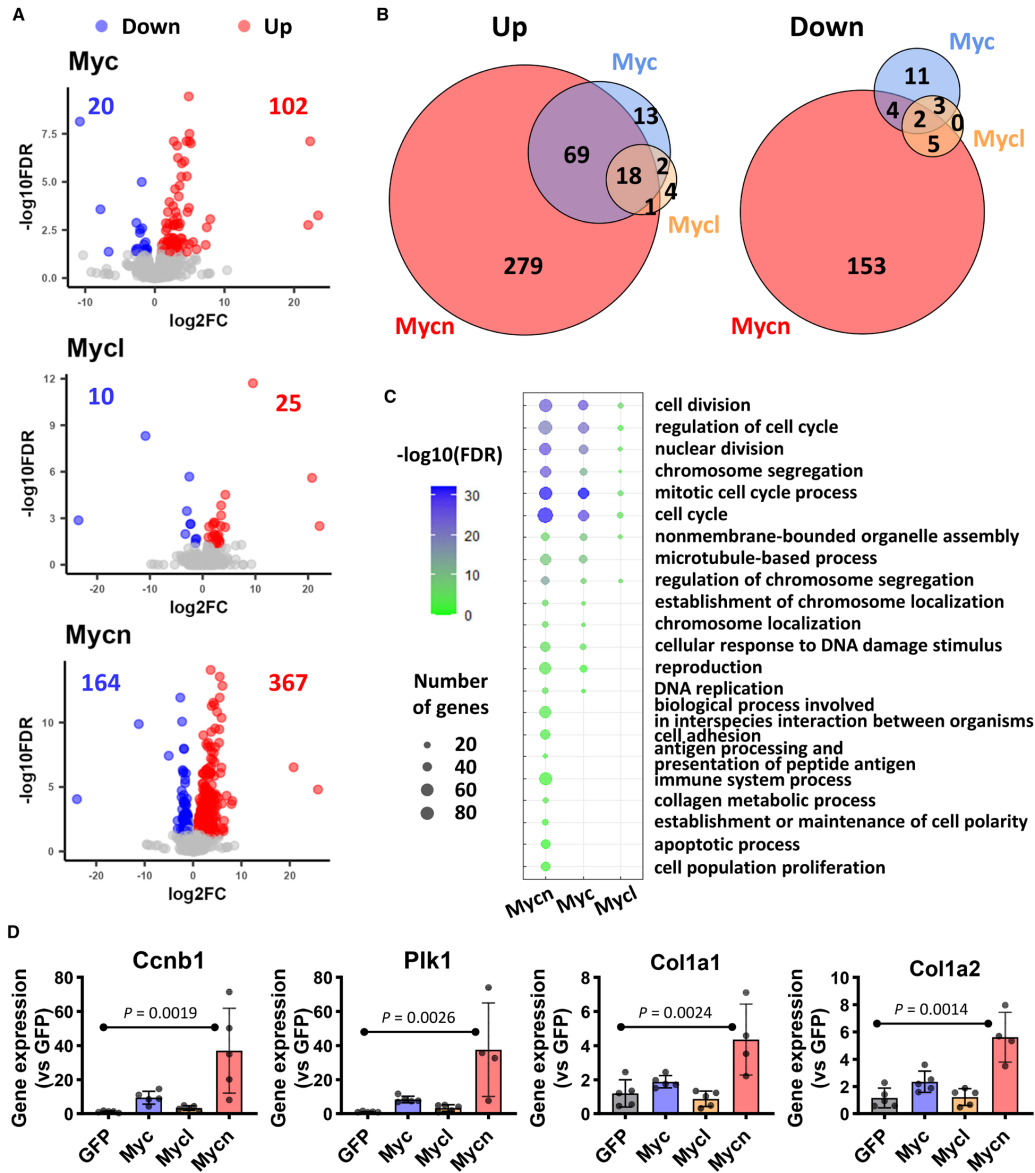
Transcriptional impact of *Myc* family gene expression in cardiomyocytes. **A**, Differential gene expression: RNA‐seq was used to identify differentially expressed genes relative to the *GFP* control group. Differential expression was analyzed using DESeq2, and genes were defined as differentially expressed when the FDR <0.05 and an absolute FC ≥2. Numbers of upregulated and downregulated genes are shown in red and blue, respectively. **B**, Venn diagram: The numbers of commonly and uniquely altered genes among the groups are indicated. **C**, GO enrichment analysis: Enriched GO terms in biological processes for upregulated genes are shown. **D**, Validation of RNA‐seq results: Quantitative reverse transcription‐polymerase chain reaction was performed for selected genes involved in G2/M phase progression and collagen metabolism. Dots represent individual biological replicates. Statistical analyses were performed using 1‐way ANOVA followed by Tukey's post hoc test. FC indicates fold change; FDR, false discovery rate; GO, Gene Ontology; and RNA‐seq, RNA sequencing.

To determine whether there are functional differences among *Myc* family members, RNA sequencing was performed on purified cardiomyocytes 2 weeks after AAV9‐mediated *Myc* family gene delivery. Differentially expressed gene analysis revealed that *Myc*, *Mycl*, and *Mycn* upregulated 102, 25, and 367 genes and downregulated 20, 10, and 164 genes, respectively (Figure 2A). The Venn diagram indicated a strong overlap in upregulated genes, with ~80% of the upregulated genes in the *Myc* and *Mycl* groups included in the upregulated genes in the *Mycn* group (Figure 2B). In contrast, although the total number of downregulated genes in the *Myc* group was smaller than in the *Mycn* group, 70% of the downregulated genes in the *Myc* group did not overlap with those in the *Mycn* group. GO enrichment analysis revealed that all *Myc* family members upregulated genes related to cell cycle regulation, with lower false discovery rates and higher gene numbers in the *Mycn* group compared with the *Myc* and *Mycl* groups. This includes GO terms such as “cell division (GO:0051301)” and “cell cycle (GO:0007049)”. Additionally, the *Mycn* group showed unique GO term enrichment, such as “collagen metabolic process (GO:0032963)” (Figure 2C; Table S3). These data indicate that although all *Myc* family members commonly activate cell cycle‐related genes, *Mycn* has the most significant impact and unique effects, particularly influencing the reorganization of the surrounding tissue environment, including cell adhesion and collagen production. Consistent with the RNA‐seq data, reverse transcription‐qPCR demonstrated that *Mycn* significantly upregulated G2/M and mitotic cell cycle regulator genes (*Ccnb1* and *Plk1*) and collagen genes (*Col1a1* and *Col1a2*) (Figure 2D).

### *Mycn* Induces Mitosis and Hypertrophy in Cardiomyocytes

**Figure 3:**
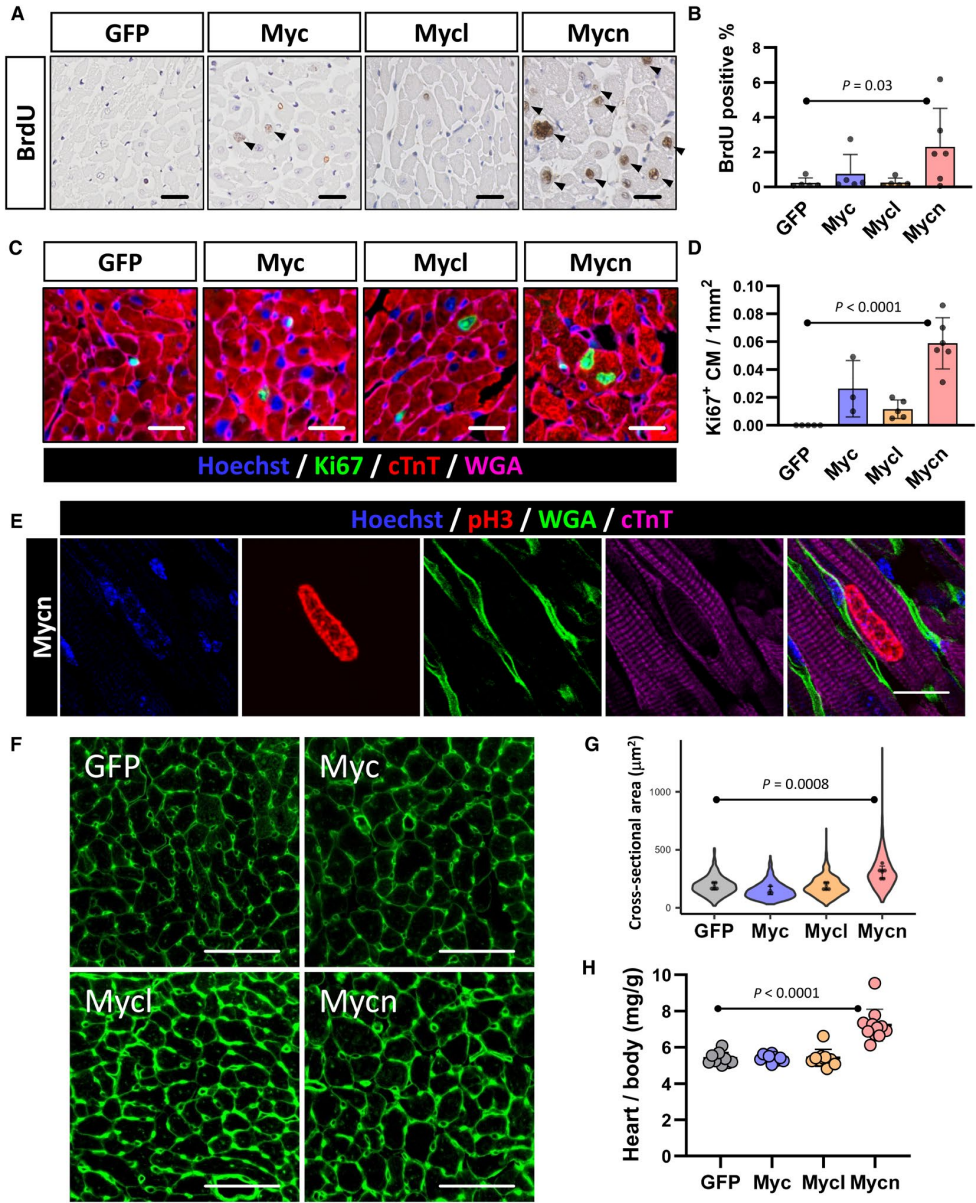
*Myc* family gene effects on cardiomyocyte cell cycle activity and cardiac growth. **A**, BrdU staining: BrdU immunostaining was performed 2 weeks after adeno‐associated virus vector administration (*GFP*, *Myc*, *Mycl*, *Mycn*). Cardiomyocyte nuclei positive for BrdU are indicated by arrowheads. Scale bar: 20 μm. **B**, Quantification of cycling nuclei: percentages of BrdU‐positive nuclei were quantified using QuPath software and include both cardiomyocyte and noncardiomyocyte nuclei. Statistical analysis was performed using 1‐way ANOVA followed by Tukey's post hoc test. **C**, Identification of Ki67‐positive cardiomyocytes: Ki67 was co–immunostained with cTnT. Ki67 is in green, cTnT in red, Hoechst in blue, and WGA in magenta. Scale bar: 20 μm. **D**, Quantification of Ki67‐positive cardiomyocyte nuclei: Ki67‐positive nuclei costained with cTnT were counted and normalized to myocardial area (mm^2^). Statistical analysis was performed using 1‐way ANOVA followed by Tukey's post hoc test. **E**, Identification of pH3 (Ser10)–positive cardiomyocytes: pH3 immunostaining was performed with cTnT. A representative image from the *Mycn* group is shown; pH3‐positive cardiomyocytes were not detected in other groups. pH3 is in red, cTnT in magenta, Hoechst in white, and WGA in green. Scale bar: 20 μm. **F**, Representative images of cardiac cross‐sections: Representative WGA‐stained sections used to assess cardiomyocyte cross‐sectional area. Scale bar: 50 μm. **G**, Quantification of cardiomyocyte cross‐sectional area: cross‐sectional areas of 100 cardiomyocytes per mouse were measured. Dots represent average values for biological replicates; violin plots depict distribution; error bars indicate SD. Statistical analysis was performed using 1‐way ANOVA followed by Tukey's post hoc test. **H**, Cardiac growth: heart weight normalized to body weight was assessed. Dots represent individual biological replicates. Statistical analysis was performed using 1‐way ANOVA followed by Tukey's post hoc test. BrdU indicates 5‐bromo‐2′‐deoxyuridine; cTnT, cardiac troponin T; pH3, phospho‐histone H3; and WGA, wheat germ agglutinin.

As gene expression analysis showed that all *Myc* family members upregulated cell cycle genes, we next examined whether *Myc* family members affected cell cycle activity and heart growth. BrdU, a thymidine analog, was administered in drinking water for 2 weeks after AAV delivery to label cardiomyocytes and noncardiomyocytes entering S phase (Figure 1A). BrdU‐positive rates in Figure 3B represent total BrdU‐positive nuclei within the myocardium. BrdU‐positive nuclei were absent or rare in the *GFP* group, with only minimal increases in the *Myc* and *Mycl* groups. In contrast, *Mycn* markedly increased BrdU incorporation, indicating S‐phase entry in both cardiomyocytes and noncardiomyocytes (3.8%±1.1% in *Mycn* versus 0.16%±0.06% in *GFP*, 0.29%±0.04% in *Myc*, and 0.55%±0.38% in *Mycl*; Figure 3A and 3B).

To identify cardiomyocytes specifically, Ki67 immunofluorescence staining was performed. Ki67‐positive cardiomyocytes were undetectable in *GFP* hearts; occasional positive cells were observed in *Myc* and *Mycl* groups, whereas *Mycn* induced a significant increase (0.06±0.01 nuclei/mm^2^ in *Mycn* versus 0.03±0.02 in *Myc* and 0.01±0.01 in *Mycl*; Figure 3C and 3D; Figure S1). Additionally, pH3‐positive cardiomyocytes were detected at low frequency only in the *Mycn* group (Figure 3E).

Despite increased cell cycle entry, evidence of completed division was limited. AurkB (aurora kinase B) immunostaining revealed no AurkB‐positive midbodies in *Mycn*‐expressing hearts (data not shown), indicating rare progression to cytokinesis. Consistent with this, RNA‐seq and qPCR demonstrated significant upregulation of cell cycle inhibitors *Cdkn1a* (p21) and *Cdkn3* (Figure S2), which may antagonize efficient cell cycle progression and restrict cardiomyocyte proliferation.

Cell cycle activation is associated with increased cell size. To measure the cardiomyocyte size, wheat germ agglutinin staining was performed (Figure 3F and 3G). The cross‐sectional area of cardiomyocytes in the *GFP* group was 188±79 μm^2^, with no significant difference observed in the *Myc* (149±72 μm^2^) and *Mycl* (185±84 μm^2^) groups. In contrast, cardiomyocyte cell size in the *Mycn* group (307±141 μm^2^) was significantly larger than in the *GFP*, *Myc*, and *Mycl* groups (Figure 3G). Consistent with the increase in cell cycle activity and cell size, heart mass normalized to body weight was significantly increased in the *Mycn* group (7.2±0.84 mg/g) compared with the *GFP*, *Myc*, and *Mycl* groups (5.4±0.30, 5.40±0.18, 5.41±0.45 mg/g, respectively; Figure 3H). Because definitive evidence of cardiomyocyte proliferation was not observed, the increase in heart mass likely reflects hypertrophic growth rather than completed cell division. In summary, *Mycn*—unlike *Myc* or *Mycl*—most strongly promotes cardiomyocyte cell cycle re‐entry and induces hypertrophy but does not drive full cardiomyocyte proliferation.

### Cardiac‐Specific *Mycn* Expression Activates Fibroblasts While Preserving Cardiac Function

**Figure 4:**
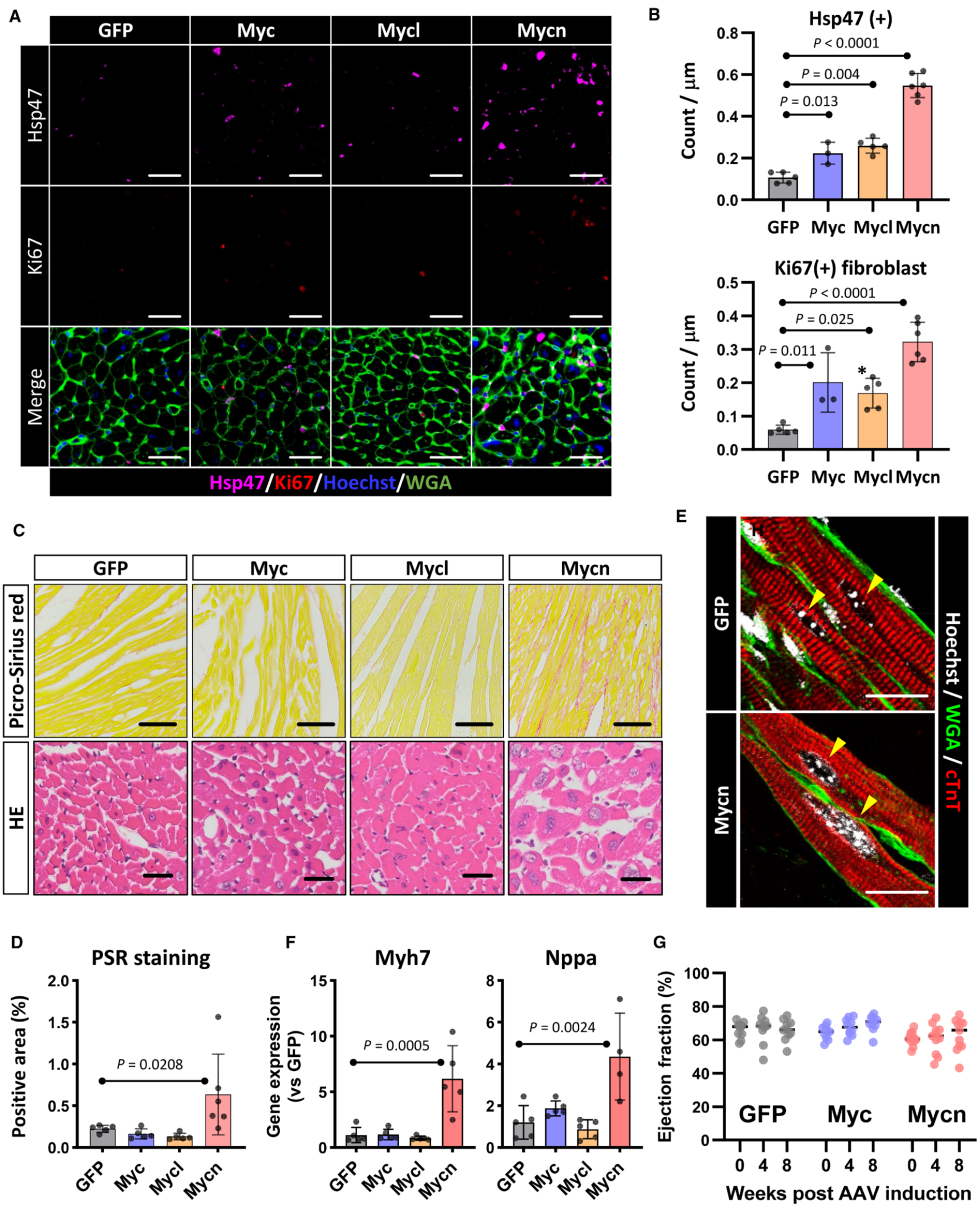
*Myc* family gene effects on fibroblast activation and cardiac phenotype. **A**, Fibroblast activation markers: immunostaining for Hsp47 (magenta) and Ki67 (red) was performed to assess fibroblast activation 2 weeks after AAV (*GFP*, *Myc*, *Mycl*, or *Mycn*) administration. WGA (green) was used to delineate cardiomyocytes. Scale bar: 30 μm. **B**, Quantification of fibroblast activation markers: numbers of Hsp47‐positive (**top**) and Ki67‐positive (**bottom**) fibroblasts per myocardial area were quantified. Statistical analyses were performed using 1‐way ANOVA followed by Tukey's post hoc test. **C**, Histological assessment: representative images of PSR (**top**) and HE (**bottom**) staining. Scale bars: 100 μm (PSR) and 25 μm (HE). **D**, Quantification of collagen production: PSR‐positive areas were quantified using QuPath software. Statistical analysis was performed by 1‐way ANOVA followed by Tukey's post hoc test. **E**, Visualization of heterochromatin: DNA was stained with Hoechst 33342 to visualize heterochromatin within cardiomyocytes. Cardiomyocyte nuclei were identified by co‐immunostaining for cTnT and WGA. Hoechst are in white, WGA in green, and cTnT in red; yellow arrowheads denote cardiomyocyte nuclei. Scale bar: 20 μm. **F**, Fetal gene expression: reverse transcription polymerase chain reaction analysis of *Myh7* and *Nppa* expression. Statistical analyses were performed using 1‐way ANOVA followed by Tukey's post hoc test. **G**, Cardiac function: ejection fraction was assessed by transthoracic echocardiography at 0, 4, and 8 weeks following AAV transduction (*GFP*, *Myc*, *Mycn*). AAV indicates adeno‐associated virus; cTnT, cardiac troponin T; HE, hematoxylin–eosin; PSR, picrosirius red; and WGA, wheat germ agglutinin.

We next assessed whether there were any pathophysiological changes in the hearts expressing *Myc* family members. Gene expression analysis indicated that *Mycn* upregulated genes related to the collagen metabolic process, in addition to cell cycle regulatory genes (Figure 2). Therefore, we first investigated whether the expression of cardiac‐specific *Myc* family members alters fibroblast activation. Hsp47 (heat shock protein 47) is a chaperone in collagen metabolism and a marker of myofibroblast activation.^22^ Immunofluorescence staining showed a significant increase in Hsp47‐positive fibroblasts in *Mycn*‐expressing heart sections (0.55±0.053 cells/mm^2^) compared with all other groups (*GFP*: 0.11±0.024 cells/mm^2^, *Myc*: 0.22±0.043 cells/mm^2^, *Mycl*: 0.26±0.031 cells/mm^2^) (Figure 4A and 4B). Similarly, the number of fibroblasts positive for Ki67, a proliferation marker, was significantly increased in the *Mycn* group (*GFP*: 0.059±0.012 cells/mm^2^, *Myc*: 0.20±0.073 cells/mm^2^, *Mycl*: 0.17±0.040 cells/mm^2^, *Mycn*: 0.32±0.054 cells/mm^2^). Consistent with these data, picrosirius red staining demonstrated a significant increase in interstitial collagen fibers, functioning as a structural framework between cardiomyocytes, in the *Mycn* group relative to the other groups (*GFP*: 0.22%±0.038%, *Myc*: 0.16%±0.054%, *Mycl*: 0.13%±0.035%, *Mycn*: 0.63%±0.44%) (Figure 4C top panel and Figure 4D). Some noticeable morphological changes were observed in the hematoxylin and eosin stained images. An increase in nuclear size and a speckled pattern of heterochromatic foci were observed in *Mycn*‐expressing cardiomyocytes (Figure 4C lower panel). These nuclear changes were confirmed by Hoechst 33342 DNA staining (Figure 4E; Figure S3).^23^ The observed heterochromatin alterations are likely associated with cell cycle activation, as previously reported.^24^ Next, qPCR was performed to assess fetal gene expression, which is often related to pathological changes and activation of the cell cycle in cardiomyocyte.^25^, ^26^ *Myh7* (*Mycn*: 6.2±2.7 fold, *GFP*: 1.1±0.6 fold, *Myc*: 1.2±0.42 fold, *Mycl*: 0.85±0.16 fold) and *Nppa* (*Mycn*: 4.2±2.3 fold, *GFP*: 1.1±0.4 fold, *Myc*: 0.82±0.4 fold, *Mycl*: 0.55±0.38 fold) gene expressions were significantly upregulated in the *Mycn* group compared with the other groups (Figure 4F). Finally, echocardiograms were performed to determine if the *Myc* family affect cardiac function, focusing on *Myc* and *Mycn*. As indicated in Figure 4G, neither *Myc* nor *Mycn* changed the ejection fraction up to 8 weeks after gene transduction, and no differences were observed compared with the *GFP* control group. These data indicate that although *Mycn* changes fibroblast activity and cardiac fetal gene expression, *Mycn*‐expressing hearts do not exhibit apparent impairment of heart function up to 8 weeks posttransgene induction.

### *Mycn* Preserves Heart Function After MI

**Figure 5:**
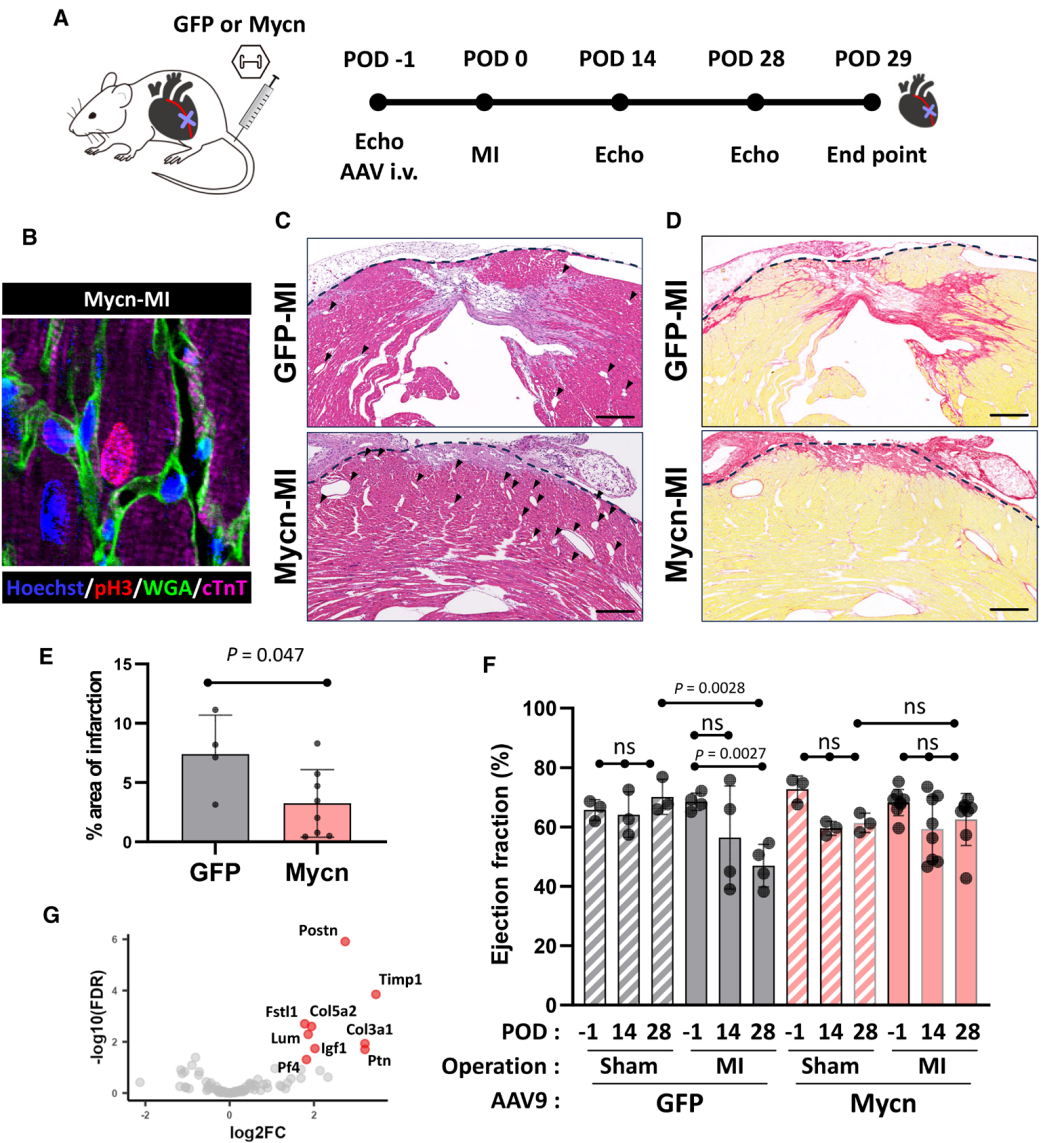
*Mycn* preserves cardiac function after myocardial infarction. **A**, Experimental design: Transthoracic echocardiography and AAV (*GFP* or *Mycn*) vector administration were performed 1 d before MI surgery. Post‐MI transthoracic echocardiography was performed on D 14 and 28, with histological analyses conducted on D 29. **B**, Identification of pH3 (Ser10)–positive cardiomyocytes: Immunostaining for pH3 was performed in combination with the cardiomyocyte marker cTnT. A representative image from the *Mycn* group at 4 weeks post–MI is shown. No pH3‐positive cardiomyocytes were detected in the *GFP* group. pH3 is in green, cTnT in red, Hoechst in blue, and WGA in magenta. Scale bar: 20 μm. **C** and **D**, Representative images of post‐MI histology: Hematoxylin–eosin (**B**) and picro‐sirius red (C) staining show myocardial architecture 28 d post–MI. Dashed lines indicate myocardial borders; arrowheads indicate vasculature in (**B**). Scale bars: 250 μm. **E**, Quantification of fibrosis: fibrotic areas were measured using QuPath software. Statistical analysis was performed using an unpaired 2‐tailed Student's *t* test. **F**, Cardiac function after MI: Ejection fraction was measured by thoracic echocardiography. Dots represent individual data points. Statistical analysis was performed using 1‐way ANOVA followed by Tukey's post hoc test. **G**, Growth factor and angiogenic gene expression: RNA‐seq data were reanalyzed to assess growth factor and angiogenesis‐related genes. Each dot represents an individual growth or angiogenic factor. Differential expression was determined using DESeq2, with significance defined as a FDR <0.05 and an absolute FC ≥2. Significantly upregulated genes are highlighted in red. AAV indicates adeno‐associated virus; cTnT, cardiac troponin T; Echo, echocardiogram; FC, fold change; FDR, false discovery rate; i.v., intravenous; MI, myocardial infarction; ns, not significant; pH3, phospho‐histone H3; POD, postoperative day; and WGA, wheat germ agglutinin.

Cycling cardiomyocytes have been reported to play a cardioprotective role.^9^ In contrast, cardiac fibroblasts can exert either cardioprotective effects by supporting tissue repair or contribute to adverse remodeling and progression of heart failure, depending on the context. Given that *Mycn* expression in cardiomyocytes had the greatest biological impact on heart tissue, we conducted further experiments to investigate the effects of *Mycn* expression on the injured myocardium. To this end, an MI model was employed on mice, which was induced via surgical ligation of the LAD. One day before MI surgery, echocardiography was performed to evaluate basal cardiac function, and AAV9 vectors (*GFP* or *Mycn*) were administered (Figure 5A). Cardiac function was assessed at 2 and 4 weeks after MI, and hearts were collected for histology at 4 weeks. To evaluate *Mycn*‐mediated cell cycle activation in the MI setting, pH3 immunofluorescence staining was performed. Consistent with baseline findings (Figure 3), pH3‐positive cardiomyocytes were readily detected in the *Mycn* group under both sham and MI conditions but were absent in *GFP* controls (Figure 5B; Figure S4). These results indicate that *Mycn* induces cardiomyocyte cell cycle activity even after MI. Histological evaluation at 4 weeks after MI revealed extensive scar formation spanning the full thickness of the left ventricular wall in the *GFP*‐expressing control group. In contrast, the *Mycn*‐expressing group exhibited only a small, superficial scar confined to the subepicardial region (Figure 5C through 5E). Notably, the myocardium in the *Mycn*‐expressing hearts showed increased vascular density in the peri‐scar area, suggesting the formation of collateral vessels (Figure 5C). In the *GFP* group, consistent with the large scar formation, ejection fraction was significantly decreased between 2 and 4 weeks after MI compared with before MI (∆ejection fraction −21.6%±7.1%). In contrast, ejection fraction was maintained through 4 weeks in the *Mycn* group after MI (∆ejection fraction −5.7%±10.9%) (Figure 5F). These data indicate that cardiac‐specific *Mycn* expression prevented the decline in heart function after MI.

Next, we explored the molecular mechanisms of cardiac protection by *Mycn*. The data showing a higher density of vasculature after MI (Figure 5C) and fibroblast activation in heart tissue (Figure 4A and 4B) by cardiac‐specific *Mycn* expression suggested that changes in the cardiac microenvironment caused cardiac protection. Therefore, to find factors affecting the cardiac microenvironment, RNA‐seq data were reanalyzed focusing on 95 genes having GO terms of “growth factor activity (GO:0008083)” or in the angiogenesis gene set.^27^ Of these, 9 genes are significantly upregulated in the *Mycn* group. Strikingly, several genes have been identified to be involved in cardioprotection (*Fstl1*, *Timp1*, *Igf1*), angiogenesis (*Fstl1*, *Igf1*, *Ptn*), and fibroblast activation (*Postn*, *Col5a2*, *Col3a1*).^28^, ^29^, ^30^, ^31^, ^32^, ^33^, ^34^ These data suggest that *Mycn*‐expressing cardiomyocytes modulated the cardiac microenvironment to resist irreversible cardiac injury, such as cardiomyocyte necrosis and myocardial tissue scarring.

## DISCUSSION

In this study, we systematically investigated the functional effects of *Myc* family genes—*Myc*, *Mycl*, and *Mycn*—in adult murine cardiomyocytes using AAV‐mediated cardiomyocyte‐specific gene delivery. Our data demonstrate that among these paralogs, *Mycn* elicits the most pronounced biological effects under identical experimental conditions, particularly in promoting cardiomyocyte cell cycle activation and modulating the cardiac microenvironment, such as fibroblast responses. Furthermore, in a murine MI model, *Mycn* expression in cardiomyocytes preserved systolic function and significantly reduced infarct area, suggesting a potent cardioprotective role. To our knowledge, this is the first study to directly compare the functional effects of the *Myc* family genes in the postnatal mammalian heart under ischemic stress, specifically MI. Our findings position *Mycn* as a potent modulator for enhancing cardioprotection through cardiomyocyte response and stromal remodeling during ischemic cardiac stress.

### Functional Divergence of *Myc* Family Members in Cardiomyocytes

Although the *Myc* family members share highly conserved basic helix–loop–helix‐leucine zipper domains, they are not functionally redundant across tissues and developmental contexts.^11^, ^12^ Consistent with this concept, we observed differential cellular responses in postnatal cardiomyocytes following expression of individual *Myc* family members under identical experimental conditions. Prior studies have shown that *Myc* overexpression in adult cardiomyocytes induces cardiac hypertrophy and DNA synthesis but fails to elicit full mitotic re‐entry, limiting its regenerative potential.^15^, ^16^, ^35^, ^36^ Our findings align with these observations, as *Myc* promoted S‐phase re‐entry but did not induce mitosis as indicated by the absence of pH3‐positive cardiomyocytes. By contrast, *Mycn* demonstrated the ability to drive both S‐phase re‐entry and mitotic progression in postnatal cardiomyocytes, as evidenced by significantly increased BrdU incorporation and pH3‐positive nuclei (Figure 3A and 3B). These findings are consistent with its developmental role, as *Mycn* is essential for cardiomyocyte proliferation during cardiac embryogenesis and regeneration. Genetic ablation of *Mycn* during embryonic development results in myocardial hypoplasia via downregulation of key cell cycle regulators such as *Ccnd1* and *Ccnd2*, whereas *Myc* is dispensable in this context.^13^, ^14^ Moreover, the Hedgehog–Gli1–Mycn signaling axis has been implicated in cardiac regeneration in both zebrafish and neonatal mice.^37^ Our results extend these developmental insights into the postnatal heart, demonstrating that *Mycn* reactivates robust cell cycle activity in postnatal cardiomyocytes, evidenced by both S and M phase progression. Collectively, these data demonstrate that *Mycn* expression is sufficient to induce cardiomyocyte cell cycle activity in the postnatal heart under the experimental conditions used in this study.

In addition, *Mycn* expression conferred cardioprotection against ischemic injury. Notably, a recent study reported that induction of *Myc* using a Myh6‐Cre–driven Rosa26 knock‐in model reduces scar size following MI.^38^ In that model, *Myc* expression is initiated during cardiac development and maintained constitutively throughout postnatal life, which differs fundamentally from our AAV‐based approach that acutely induces *Myc* family gene expression in mature cardiomyocytes. Despite these differences, both models exhibit cardioprotective phenotypes, suggesting that *Myc* family members may exert overlapping functions in the heart depending on experimental context. Further studies systematically varying expression levels and duration will be required to delineate shared versus isoform‐specific roles among *Myc* family members.

### Potential Mechanisms of *Mycn*‐Mediated Cardioprotection

In this study, we demonstrated that *Mycn*‐expressing postnatal hearts exhibit significantly preserved systolic function following MI—which we associate with reduced infarct area and increased capillary density in the peri‐infarct region (Figure 5C and 5D). Cardiomyocyte‐intrinsic changes alone are unlikely to account for the full extent of beneficial post‐MI tissue remodeling observed in these *Mycn*‐expressing hearts. Although detailed mechanisms remain for future investigation, our transcriptomic analysis revealed that *Mycn* robustly upregulated a suite of genes associated with paracrine signaling, extracellular matrix remodeling, and stromal activation—many of which are enriched in neonatal cardiac environments, including *Fstl1*, *Timp1*, *Igf1*, *Postn*, *Ptn*, *Pf4*, *Col5a2*, *Col3a1*, and *Lum*—known to enhance angiogenesis, suppress inflammation, or promote cardiomyocyte survival under ischemic stress.^28^, ^29^, ^30^, ^31^, ^32^, ^33^, ^34^ For instance, *FSTL1* is a known secreted factor that promotes angiogenesis and cardiomyocyte survival after MI^28^, ^29^; *Timp1* stabilizes the extracellular matrix and prevents adverse remodeling, and its deletion exacerbates cardiac dysfunction after myocardial injury^30^, ^31^; *IGF1* enhances cardiomyocyte survival via the PI3K/Akt pathway and supports cardiomyocyte metabolic adaptation during ischemic stress.^32^, ^33^ These factors secreted from *Mycn*‐expressing cardiomyocytes may collectively contribute to constructing a cardioprotective microenvironment enhancing tissue resilience against irreversible ischemic injury.

In addition, we found that *Mycn*‐expressing murine hearts exhibited increased numbers of Hsp47‐positive fibroblasts and elevated collagen production, as shown by picrosirius red staining (Figure 4A through 4D). Traditionally, fibroblast activation and collagen deposition are hallmarks of adverse remodeling and cardiac fibrosis.^22^, ^39^, ^40^ However, emerging studies have identified functionally distinct fibroblast subtypes—including proangiogenic, immunomodulatory, and extracellular‐matrix‐stabilizing phenotypes—each responsive to paracrine cues from neighboring cardiomyocytes; notably, *Hsp47* itself is essential for collagen maturation and is essential to prevent ventricular rupture during acute phases of cardiac injury.^41^, ^42^, ^43^ This study showed absence of cardiac dysfunction at up to 8 weeks, and preserved ejection fraction after MI in *Mycn*‐expressing hearts, raising the possibility that fibroblast activation in this setting may include reparative or protective subtypes distinct from classical pathological fibroblasts. However, the heterogeneity and functional significance of activated fibroblasts in *Mycn*‐expressing hearts remain unresolved, and additional mechanistic studies will be required to determine whether distinct fibroblast subpopulations with adaptive, reparative, or pathological roles are present.

These mechanistic interpretations remain correlative, as they are based on transcriptomic and histological observations and require direct experimental validation. Future studies incorporating targeted perturbation of candidate paracrine factors, fibroblast‐specific transcriptomic profiling, and mechanistic assays in both in vivo and in vitro systems will be essential to determine whether and how *Mycn*‐expressing cardiomyocytes regulate fibroblast behavior and mediate cardioprotection. Such approaches will clarify causal relationships underlying *Mycn*‐induced phenotypes and define the precise mechanisms by which *Mycn* modulates the myocardial microenvironment.

### Correlation Between Cell Cycle Activation and Cardioprotection After MI

Although direct mechanisms remain uninvestigated, the correlation between *Mycn* induced robust cell cycle re‐entry and cardioprotection following MI observed in our study aligns with Bradley and colleagues' recent findings—that existence of cycling cardiomyocytes correlates with cardioprotection from ischemic injury.^9^ One potential explanation is that cell cycle activity serves as a surrogate marker of a broader phenotypic shift toward a fetal‐like state of plasticity, characterized by altered gene expression profiles that confer injury resistance. To reinforce this idea, many of the *Mycn*‐induced genes already discussed are abundantly expressed in naturally regenerative neonatal cardiomyocytes (Figure S5A). Furthermore, in regenerative cardiac models (via YAP [Yes‐associated protein] activation or transient OSKM [Oct4, Sox2, Klf4 and c‐Myc] induction), cardiomyocytes acquire fetal‐like transcriptional signatures, acquiring enhanced stress tolerance and regenerative capacity.^44^, ^45^—our data show that more than half of *Mycn*‐induced genes overlapped with those highly expressed in neonatal cardiomyocytes (Figure S5B), supporting the concept of fetal‐like phenotype shift in postnatal cardiomyocytes. Thus, the correlation between cardiomyocyte cell cycle activity and cardiac protection may not reflect a direct causal relationship, but rather a phenotypic shift toward a fetal‐like stress‐adaptive state; this altered state may orchestrate cardioprotection through paracrine signaling and modulation of fibroblast phenotypes, ultimately constructing a supportive cardiac microenvironment.

## LIMITATIONS

Although *Myc* family transgenes were robustly overexpressed at the mRNA level (Figure 1) and induced clear biological effects in cardiomyocytes (Figures 2 through 5), several technical limitations warrant acknowledgment. First, quantitative assessment of transgene protein abundance and spatial distribution was not achieved. Immunofluorescence staining with an anti‐V5 antibody failed to detect GFP (green fluorescent protein) or Myc family proteins, likely due to their intrinsically low abundance and limited antibody sensitivity. Consistently, Western blotting detected GFP but not Myc, Mycl, or Mycn (Figure S6). The markedly lower abundance of Myc family proteins relative to GFP likely reflects posttranslational regulation, as Myc proteins undergo rapid proteasomal degradation and are typically maintained at low levels in normal cells.^46^

To address this limitation, we used anti‐GFP immunofluorescence as a surrogate for AAV9 transduction. Although this approach cannot substitute for direct quantification of Myc family proteins, the evenly distributed GFP signal (Figure S7) suggests comparable transgene delivery across constructs, given that all AAV vectors shared the same backbone and promoter. This inference is further supported by similar mRNA expression levels measured using woodchuck hepatitis virus posttranscriptional regulatory element ‐based qPCR (Figure 1B).

Despite these constraints, the identical experimental framework revealed a distinct and robust biological activity of *Mycn* compared with *Myc* and *Mycl* in adult cardiomyocytes. Nonetheless, the inability to directly quantify Myc family protein expression and localization remains a limitation. Future studies employing more sensitive reporter systems, alternative epitope‐tagging strategies, or dose‐dependent expression analyses will be essential to define protein‐level expression and elucidate the mechanisms underlying *Mycn*'s unique functional effects.

## CONCLUSIONS

In summary, this study demonstrates that *Mycn* activates cardiomyocyte cell cycle re‐entry and confers significant cardioprotection following MI in the postnatal murine heart. This protective effect is likely mediated, at least in part, by reactivation of a fetal‐like cardiomyocyte state, which coordinates stress‐adaptive responses across cardiomyocytes and stromal cells alike. Our findings enhance the understanding of cardiac plasticity and support the therapeutic potential of *Mycn* or its downstream effectors in promoting cardiac repair in hearts under ischemic stress.

## Sources of Funding

This work was supported by Japan Society for the Promotion of Science Grants‐in‐Aid for Scientific Research (Grant Number 23K08225 to Aina Hirofuji; and Grant Number 21K08854, and 24K02526 to Kyohei Oyama), Japan Surgical Society (Young Researcher Award 2023–2024 to Aina Hirofuji), and Japan Society for the Promotion of Science Bilateral Joint Research Project (Grant Number JPJSBP12023990 to Kyohei Oyama).

## Disclosures

None.

## Supporting information

Data S1–S2
Tables S1–S3
Figures S1–S7

Unedited Blots
