# Supplementary material for: Mycn Reactivates the Cell Cycle in Adult Cardiomyocytes and Promotes Cardioprotection in Myocardial Infarction
Source: J Am Heart Assoc. 2026 Apr 9;15(8):e046146. doi: 10.1161/JAHA.125.046146 (PMC13279142; doi:10.1161/JAHA.125.046146)
Supplement: Supplementary file 1 — Data S1–S2 Tables S1–S3 Figures S1–S7 [file JAH3-15-e046146-s002.pdf]

SUPPLEMENTAL MATERIAL

Data S1. Supplemental Methods

5’ homology to back bone

ORF (GFP, Myc, Mycl, Mycn)

Linker with V5

3’ homology to back bone

GFP-V5

CTCACTATAGGCTAGGCCGCCATGGTGAGCAAGGGCGAGGAGCTGTTCACCGGGGTGGTGCCCATCCTGGTC
GAGCTGGACGGCGACGTAAACGGCCACAAGTTCAGCGTGTCCGGCGAGGGCGAGGGCGATGCCACCTACGG
CAAGCTGACCCTGAAGTTCATCTGCACCACCGGCAAGCTGCCCGTGCCCTGGCCCACCCTCGTGACCACCCTG
ACCTACGGCGTGCAGTGCTTCAGCCGCTACCCCGACCACATGAAGCAGCACGACTTCTTCAAGTCCGCCATGC
CCGAAGGCTACGTCCAGGAGCGCACCATCTTCTTCAAGGACGACGGCAACTACAAGACCCGCGCCGAGGTGA
AGTTCGAGGGCGACACCCTGGTGAACCGCATCGAGCTGAAGGGCATCGACTTCAAGGAGGACGGCAACATC
CTGGGGCACAAGCTGGAGTACAACTACAACAGCCACAACGTCTATATCATGGCCGACAAGCAGAAGAACGGC
ATCAAGGTGAACTTCAAGATCCGCCACAACATCGAGGACGGCAGCGTGCAGCTCGCCGACCACTACCAGCAG
AACACCCCCATCGGCGACGGCCCCGTGCTGCTGCCCGACAACCACTACCTGAGCACCCAGTCCGCCCTGAGCA
AAGACCCCAACGAGAAGCGCGATCACATGGTCCTGCTGGAGTTCGTGACCGCCGCCGGGATCACTCTCGGCAT
GGACGAGCTGTACAAGTCAGGCCTCATGGGCCCAGCTTTCTTGTACAAAGTGGTTGATATCCAGCACAGTGGC
GGCCGCTCGAGTCTAGAGGGCCCGCGGTTCGAAGGTAAGCCTATCCCTAACCCTCTCCTCGGTCTCGATTCTAC
GTGACTCTAGAGTCGACCC

Myc-V5

CTCACTATAGGCTAGGCCGCCATGCCCCTCAACGTGAACTTCACCAACAGGAACTATGACCTCGACTACGACTC
CGTACAGCCCTATTTCATCTGCGACGAGGAAGAGAATTTCTATCACCAGCAACAGCAGAGCGAGCTGCAGCCG
CCCGCGCCCAGTGAGGATATCTGGAAGAAATTCGAGCTGCTTCCCACCCCGCCCCTGTCCCCGAGCCGCCGCT
CCGGGCTCTGCTCTCCATCCTATGTTGCGGTCGCTACGTCCTTCTCCCCAAGGGAAGACGATGACGGCGGCGG
TGGCAACTTCTCCACCGCCGATCAGCTGGAGATGATGACCGAGTTACTTGGAGGAGACATGGTGAACCAGAG
CTTCATCTGCGATCCTGACGACGAGACCTTCATCAAGAACATCATCATCCAGGACTGTATGTGGAGCGGTTTCTC
AGCCGCTGCCAAGCTGGTCTCGGAGAAGCTGGCCTCCTACCAGGCTGCGCGCAAAGACAGCACCAGCCTGAG
CCCCGCCCGCGGGCACAGCGTCTGCTCCACCTCCAGCCTGTACCTGCAGGACCTCACCGCCGCCGCGTCCGAG
TGCATTGACCCCTCAGTGGTCTTTCCCTACCCGCTCAACGACAGCAGCTCGCCCAAATCCTGTACCTCGTCCGAT
TCCACGGCCTTCTCTCCTTCCTCGGACTCGCTGCTGTCCTCCGAGTCCTCCCCACGGGCCAGCCCTGAGCCCCT
AGTGCTGCATGAGGAGACACCGCCCACCACCAGCAGCGACTCTGAAGAAGAGCAAGAAGATGAGGAAGAAA
TTGATGTGGTGTCTGTGGAGAAGAGGCAAACCCCTGCCAAGAGGTCGGAGTCGGGCTCATCTCCATCCCGAG
GCCACAGCAAACCTCCGCACAGCCCACTGGTCCTCAAGAGGTGCCACGTCTCCACTCACCAGCACAACTACGC

CGCACCCCCCTCCACAAGGAAGGACTATCCAGCTGCCAAGAGGGCCAAGTTGGACAGTGGCAGGGTCCTGAA
GCAGATCAGCAACAACCGCAAGTGCTCCAGCCCCAGGTCCTCAGACACGGAGGAAAACGACAAGAGGCGGA
CACACAACGTCTTGGAACGTCAGAGGAGGAACGAGCTGAAGCGCAGCTTTTTTGCCCTGCGTGACCAGATCC
CTGAATTGGAAAACAACGAAAAGGCCCCCAAGGTAGTGATCCTCAAAAAAGCCACCGCCTACATCCTGTCCAT
TCAAGCAGACGAGCACAAGCTCACCTCTGAAAAGGACTTATTGAGGAAACGACGAGAACAGTTGAAACACAA
ACTCGAACAGCTTCGAAACTCTGGTGCACAGGCCTCATGGGCCCAGCTTTCTTGTACAAAGTGGTTGATATCCA
GCACAGTGGCGGCCGCTCGAGTCTAGAGGGCCCGCGGTTCGAAGGTAAGCCTATCCCTAACCCTCTCCTCGGT
CTCGATTCTACGTGACTCTAGAGTCGACCC

Mycl-V5

CTCACTATAGGCTAGGCCGCCATGGACTTCGACTCGTATCAGCACTATTTCTACGACTATGACTGCGGAGAGGAT
TTCTACCGCTCCACGGCGCCCAGCGAGGACATCTGGAAGAAATTCGAGCTGGTGCCGTCGCCCCCCACGTCGC
CGCCCTGGGGCTCCGGTCCCGGCGCCGTGGACCCAGCCTCTGGGATTAATCCCGGGGAGCCGTGGCCTGGAG
GGGGTGCCGGGGACGAGGCGGAATCTCGGGGCCATTCGAAAGCCTGGGGCAGGAATTATGCTTCCATCATTC
GCCGTGACTGCATGTGGAGCGGCTTCTCCGCCCGAGAACGGCTGGAGAGAGTGGTGAGCGACAGGCTGGCC
CCAGGCGCGCCCCGGGGGAACCCGCCCAAAGCGCCCGCTACCCCGGACGGCACTCCTAGTCTGGAAGCCAG
TAACCCGGCGCCCGCCACCCAATGTCAGCTGGGCGAGCCCAAGACTCAGGCCTGCTCCGGGTCCGAGAGCCC
CAGCGATTCTGAAGGTGAAGAGATTGACGTGGTGACCGTGGAGAAGAGGCGATCTCTGGACATCCGAAAGCC
AGTCACCATCACGGTGCGAGCAGACCCCCTGGACCCCTGCATGAAGCACTTCCATATCTCTATCCACCAACAGC
AGCATAACTATGCTGCCCGTTTTCCTCCAGAAAGTTGCTCTCAAGAGGGGGATCCTGAGCCAGGTCCCCAGGA
AGAGGCTCCGGAGATAGAAGCTCCCAAGGAGAAAGAGGAGGAGGAAGAGGAAGAGGAGGAAGAAGAGAT
TGTGAGCCCCCCACCTGTCGGAAGTGAGGCTCCCCAGTCCTGCCACCCCAAACCTGTCAGTTCTGACACTGAG
GACGTGACCAAGAGGAAGAACCATAACTTCTTGGAACGAAAAAGGAGGAATGACCTCCGCTCCCGGTTCCTA
GCCCTGCGGGACCAGGTTCCCACCCTGGCCAGCTGCTCTAAGGCCCCCAAAGTCGTGATCCTCAGCAAGGCGT
TAGAATACTTGCAGGCTTTGGTGGGGGCTGAAAAGAAAATGGCTACAGAGAAAAGGCAGCTCCGGTGTCGG
CAACAGCAACTGCAAAAGAGAATCGCGTACCTCAGTGGCTACCAGGCCTCATGGGCCCAGCTTTCTTGTACAA
AGTGGTTGATATCCAGCACAGTGGCGGCCGCTCGAGTCTAGAGGGCCCGCGGTTCGAAGGTAAGCCTATCCCT
AACCCTCTCCTCGGTCTCGATTCTACGTGACTCTAGAGTCGACCC

Mycn-V5

CTCACTATAGGCTAGGCCGCCATGCCCAGCTGCACCGCGTCCACCATGCCGGGGATGATCTGCAAGAACCCAG
ACCTCGAGTTTGACTCACTGCAGCCCTGCTTCTACCCGGACGAAGATGACTTCTACTTCGGCGGTCCCGACTCG
ACCCCACCGGGGGAGGACATCTGGAAGAAGTTTGAGCTGCTGCCCACGCCCCCGTTGTCGCCCAGCCGCGCC
TTCCCAGAGCACAGCCCGGAGCCTTCGAATTGGGCTACGGAGATGCTGCTGCCGGAGGCCGACCTGTGGGGC
AACCCGGCCGAGGAGGATGCGTTCGGTCTCGGGGGCCTGGGTGGCCTCACTCCTAATCCGGTCATCCTTCAGG
ACTGCATGTGGAGCGGCTTCTCTGCCCGCGAGAAGCTAGAGCGCGCAGTGAACGAAAAACTACAGCACGGCC

ACGGGCCCCCGGGCGTCAGCTCAGCCTGCTCGGCTCCCGGAGTGGGTGCCAGCAGCCCCGGGGGCCGTGCC
CTTGGTGGGTCGTCGAGTGCTAGCCACACCGGGGCCACCCTGCCTACCGACCTCTCCCACCCGGCTGCCGAAT
GTGTGGACCCCGCCGTGGTCTTCCCCTTCCCGGTGAACAAGCGAGAGTCGGCGTCGGTGCCCGCTGCCCCCA
CTAGCGCCCCGGCGACCAGCGCTGCGGTCACTAGTGTGTCTGTTCCAGCTACTGCCCCGGTGGCTGCTCCTGC
TCGTGCAGGCGGCCGTCCTGCCAGCAGTGGGGAGGCCAAGGCCCTCAGCACCTCCGGAGAGGATACCTTGA
GCGACTCAGATGATGAGGATGACGAGGAGGAAGATGAAGAGGAGGAAATCGATGTGGTCACCGTAGAGAAG
AGACGTTCCTCCTCTAACAACAAGGCGGTAACCACTTTCACGATCACTGTGCGTCCCAAGACCTCCGCTCTGGG
CCTGGGGCGAGCACAGCCTGGCGAGCTGATCCTCAAGCGCTGTGTTCCCATCCATCAGCAGCACAACTATGCT
GCACCCTCACCCTACGTGGAGAGCGAGGACGCGCCCCCGCAGAAAAAGATCAAGAGCGAGGCTTCTCCACGC
CCCCTCAAAAGTGTTGTTCCAGCAAAAGCGAAGAGCCTGAGCCCCCGAAACTCAGACTCGGAGGACAGCGA
GCGCCGCCGCAACCACAACATCCTGGAGCGTCAACGCCGGAACGACCTGCGCTCCAGCTTCCTGACGCTCAG
GGACCATGTGCCTGAGCTGGTGAAGAACGAGAAGGCCGCCAAGGTGGTCATCTTGAAAAAGGCCACCGAGT
ACGTGCACGCCCTACAGGCCAACGAGCACCAGCTCCTGCTGGAAAAGGAGAAACTGCAGGCGAGGCAGCAG
CAGTTGCTAAAGAAGATCGAACACGCTCGGACTTGCCAGGCCTCATGGGCCCAGCTTTCTTGTACAAAGTGGT
TGATATCCAGCACAGTGGCGGCCGCTCGAGTCTAGAGGGCCCGCGGTTCGAAGGTAAGCCTATCCCTAACCCT
CTCCTCGGTCTCGATTCTACGTGACTCTAGAGTCGACCC

Data S2. Primers

Target	Primer names	Sequence (5' -> 3')
**EGFP**	EGFP_F	ACCCCGACCACATGAAGCAG
	EGFP_R	TGTCGCCCTCGAACTTCACC
**Myc**	Myc_F	GTCCGATTCCACGGCCTTCT
	Myc_R	CTGTGCGGAGGTTTGCTGTG
**Mycl**	Mycl_F	CGGACGGCACTCCTAGTCTG
	Mycl_R	TTCTCCACGGTCACCACGTC
**Mycn**	Mycn_F	CTTCTCCACGCCCCCTCAAA
	Mycn_R	ACGCTCCAGGATGTTGTGGT
**Ccnb1**	Ccnb1_F	GCCTCACAAAGCACATGACTG
	Ccnb1_R	TCGACAACTTCCGTTAGCCT
**Plk1**	Plk1_F	CAGCCAGGTTCGTGGAGCAA
	Plk1_R	TCTCCCGGAACCCCACCTTT
**Col1a1**	Col1a1_F	GGTGAGACGTGGAAACCCGA
	Col1a1_R	GGGCAGAAAGCACAGCACTC
**Col1a2**	Col1a2_F	GGTGAAGCTGGCAACATCGG
	Col1a2_R	AAGACCGGGGTGTCCTCTCT
**Myh7**	Myh7_F	GCCGAGTCCCAGGTCAACAA
	Myh7_R	ACTCCTCATTCAGGCCCTTGG
**Nppa**	Nppa_F	CTTGGCCTTTTGGCTTCCAGG
	Nppa_R	GGGCAGATCTATCGGAGGGG
**Gapdh**	Gapdh_F	CCAATGTGTCCGTCGTGGATCT
	Gapdh_R	GTTGAAGTCGCAGGAGACAACC
**S26**	S26_F	GCCATCCATAGCAAGGTTGT
	S26_R	GCCTCTTTACATGGGCTTTG
**Cdkn1a**	Cdkn1a_F	GCAGACCAGCCTGACAGATTT
	Cdkn1a_R	CTGACCCACAGCAGAAGAGG
**Cdkn3**	Cdkn3_F	CGCGAGTGAATTGTTCCCAGT
	Cdkn3_R	TGGCCAGCTCCTCCATGATTT
**WPRE**	WPRE_F	CCGTTGTCAGGCAACGTG
	WPRE_R	AGCTGACAGGTGGTGGCAAT

Table S1. Comparison of permutation ANOVA and one-way ANOVA

genes	one-way ANOVA	permutation ANOVA	Note
Myc	0.0001	0.0003	Figure 1B
Mycl	0.0001	$< 10^{-4}$	Figure 1B
Mycn	0.0001	0.0001	Figure 1B
WPRE	0.2083	0.2045	Figure 1B, performed only for GFP, Myc, Mycl, Mycn
Ccnb1	0.0013	0.0005	Figure 2D
Plk1	0.0021	0.0004	Figure 2D
Col1a1	0.001	0.0002	Figure 2D
Col1a2	0.0001	0.0001	Figure 2D
Myh7	0.0001	0.0001	Figure 4F
Nppa	0.001	0.001	Figure 4F

Table S2. Pairwise comparisons of each figure

Figure	Analysis	Comparison	Mean difference	95% CI	Adjusted p-Value
1B	Myc	GFP vs. Myc	-12.87	-16.27 to -9.462	<0.0001
1B	Myc	GFP vs. Mycl	0.1806	-3.224 to 3.585	0.9987
1B	Myc	GFP vs. Mycn	-0.407	-3.812 to 2.997	0.9857
1B	Myc	Myc vs. Mycl	13.05	9.642 to 16.45	<0.0001
1B	Myc	Myc vs. Mycn	12.46	9.055 to 15.86	<0.0001
1B	Myc	Mycl vs. Mycn	-0.5877	-3.992 to 2.817	0.9593
1B	Mycl	GFP vs. Myc	0.9732	-160.0 to 161.9	>0.9999
1B	Mycl	GFP vs. Mycl	-309	-470.0 to -148.1	0.0003
1B	Mycl	GFP vs. Mycn	-0.5613	-161.5 to 160.4	>0.9999
1B	Mycl	Myc vs. Mycl	-310	-470.9 to -149.1	0.0003
1B	Mycl	Myc vs. Mycn	-1.534	-162.5 to 159.4	>0.9999
1B	Mycl	Mycl vs. Mycn	308.5	147.5 to 469.4	0.0003
1B	Mycn	GFP vs. Myc	-0.1791	-3.741 to 3.383	0.9989
1B	Mycn	GFP vs. Mycl	-0.9236	-4.486 to 2.638	0.8788
1B	Mycn	GFP vs. Mycn	-8.498	-12.06 to -4.936	<0.0001
1B	Mycn	Myc vs. Mycl	-0.7445	-4.307 to 2.818	0.9312
1B	Mycn	Myc vs. Mycn	-8.319	-11.88 to -4.757	<0.0001
1B	Mycn	Mycl vs. Mycn	-7.574	-11.14 to -4.012	<0.0001
1B	WPRE	GFP vs. Myc	0.3101	-0.8369 to 1.457	0.8653
1B	WPRE	GFP vs. Mycl	-0.5659	-1.713 to 0.5810	0.5105
1B	WPRE	GFP vs. Mycn	-0.2214	-1.368 to 0.9255	0.9446
1B	WPRE	Myc vs. Mycl	-0.8759	-2.023 to 0.2710	0.1696
1B	WPRE	Myc vs. Mycn	-0.5315	-1.678 to 0.6155	0.5607
1B	WPRE	Mycl vs. Mycn	0.3445	-0.8024 to 1.491	0.8254
2D	Ccnb1	GFP vs. Myc	-8.288	-31.14 to 14.56	0.7303
2D	Ccnb1	GFP vs. Mycl	-2.205	-25.05 to 20.64	0.9924
2D	Ccnb1	GFP vs. Mycn	-35.83	-58.68 to -12.98	0.0019
2D	Ccnb1	Myc vs. Mycl	6.083	-16.76 to 28.93	0.8703
2D	Ccnb1	Myc vs. Mycn	-27.54	-50.39 to -4.693	0.0157
2D	Ccnb1	Mycl vs. Mycn	-33.62	-56.47 to -10.78	0.0033
2D	Plk1	GFP vs. Myc	-7.328	-29.83 to 15.17	0.7849
2D	Plk1	GFP vs. Mycl	-2.336	-24.84 to 20.17	0.9903
2D	Plk1	GFP vs. Mycn	-36.47	-60.34 to -12.60	0.0026
2D	Plk1	Myc vs. Mycl	4.992	-17.51 to 27.49	0.9176
2D	Plk1	Myc vs. Mycn	-29.14	-53.01 to -5.273	0.0147
2D	Plk1	Mycl vs. Mycn	-34.13	-58.00 to -10.26	0.0045
2D	Col1a1	GFP vs. Myc	-0.674	-2.606 to 1.258	0.7487
2D	Col1a1	GFP vs. Mycl	0.326	-1.606 to 2.258	0.961
2D	Col1a1	GFP vs. Mycn	-3.156	-5.205 to -1.106	0.0024
2D	Col1a1	Myc vs. Mycl	1	-0.9322 to 2.932	0.4663
2D	Col1a1	Myc vs. Mycn	-2.482	-4.531 to -0.4321	0.0155
2D	Col1a1	Mycl vs. Mycn	-3.482	-5.531 to -1.432	0.001
2D	Col1a2	GFP vs. Myc	-1.188	-3.074 to 0.6983	0.3047
2D	Col1a2	GFP vs. Mycl	-0.064	-1.950 to 1.822	0.9996
2D	Col1a2	GFP vs. Mycn	-4.465	-6.465 to -2.464	<0.0001
2D	Col1a2	Myc vs. Mycl	1.124	-0.7623 to 3.010	0.3493
2D	Col1a2	Myc vs. Mycn	-3.277	-5.277 to -1.276	0.0014
2D	Col1a2	Mycl vs. Mycn	-4.401	-6.401 to -2.400	<0.0001

Figure	Analysis	Comparison	Mean difference	95% CI	Adjusted p-Value
3B	BrdU	GFP vs. Myc	-13.35	-73.51 to 46.81	0.9207
3B	BrdU	GFP vs. Mycl	-7.338	-67.50 to 52.82	0.9852
3B	BrdU	GFP vs. Mycn	-61.55	-119.2 to -3.952	0.034
3B	BrdU	Myc vs. Mycl	6.014	-54.15 to 66.17	0.9917
3B	BrdU	Myc vs. Mycn	-48.2	-105.8 to 9.399	0.1194
3B	BrdU	Mycl vs. Mycn	-54.21	-111.8 to 3.385	0.069
3D	Ki67(+)CM	GFP vs. Myc	-0.02633	-0.05453 to 0.001868	0.0712
3D	Ki67(+)CM	GFP vs. Mycl	-0.0116	-0.03602 to 0.01282	0.5363
3D	Ki67(+)CM	GFP vs. Mycn	-0.05883	-0.08222 to -0.03545	<0.0001
3D	Ki67(+)CM	Myc vs. Mycl	0.01473	-0.01347 to 0.04293	0.4585
3D	Ki67(+)CM	Myc vs. Mycn	-0.0325	-0.05981 to -0.005195	0.0174
3D	Ki67(+)CM	Mycl vs. Mycn	-0.04723	-0.07062 to -0.02385	0.0002
3G	CM size	GFP vs. Myc	36.26	-48.44 to 121.0	0.6161
3G	CM size	GFP vs. Mycl	-0.3794	-73.73 to 72.97	>0.9999
3G	CM size	GFP vs. Mycn	-122.1	-192.3 to -51.86	0.0008
3G	CM size	Myc vs. Mycl	-36.64	-121.3 to 48.06	0.6084
3G	CM size	Myc vs. Mycn	-158.3	-240.4 to -76.34	0.0003
3G	CM size	Mycl vs. Mycn	-121.7	-191.9 to -51.48	0.0008
3H	HW/BW	GFP vs. Myc	-0.008276	-0.6629 to 0.6463	>0.9999
3H	HW/BW	GFP vs. Mycl	-0.01884	-0.6734 to 0.6358	0.9998
3H	HW/BW	GFP vs. Mycn	-1.828	-2.467 to -1.188	<0.0001
3H	HW/BW	Myc vs. Mycl	-0.01056	-0.6652 to 0.6440	>0.9999
3H	HW/BW	Myc vs. Mycn	-1.819	-2.459 to -1.180	<0.0001
3H	HW/BW	Mycl vs. Mycn	-1.809	-2.448 to -1.169	<0.0001
4B top	Hsp47(+)	GFP vs. Myc	-0.1165	-0.2106 to -0.02251	0.0132
4B top	Hsp47(+)	GFP vs. Mycl	-0.153	-0.2344 to -0.07158	0.0004
4B top	Hsp47(+)	GFP vs. Mycn	-0.441	-0.5190 to -0.3631	<0.0001
4B top	Hsp47(+)	Myc vs. Mycl	-0.03647	-0.1305 to 0.05755	0.6845
4B top	Hsp47(+)	Myc vs. Mycn	-0.3245	-0.4155 to -0.2335	<0.0001
4B top	Hsp47(+)	Mycl vs. Mycn	-0.288	-0.3660 to -0.2101	<0.0001
4B bottom	Hsp47(+) Fib	GFP vs. Myc	-0.1418	-0.2533 to -0.03034	0.011
4B bottom	Hsp47(+) Fib	GFP vs. Mycl	-0.109	-0.2055 to -0.01248	0.0245
4B bottom	Hsp47(+) Fib	GFP vs. Mycn	-0.263	-0.3554 to -0.1706	<0.0001
4B bottom	Hsp47(+) Fib	Myc vs. Mycl	0.0328	-0.07866 to 0.1443	0.8308
4B bottom	Hsp47(+) Fib	Myc vs. Mycn	-0.1212	-0.2291 to -0.01325	0.0254
4B bottom	Hsp47(+) Fib	Mycl vs. Mycn	-0.154	-0.2464 to -0.06155	0.0012
4D	PSR	GFP vs. Myc	0.0617	-0.2928 to 0.4162	0.718
4D	PSR	GFP vs. Mycl	0.0938	-0.2607 to 0.4483	0.584
4D	PSR	GFP vs. Mycn	-0.41	-0.7494 to -0.07053	0.0208
4D	PSR	Myc vs. Mycl	0.0321	-0.3224 to 0.3866	0.8508
4D	PSR	Myc vs. Mycn	-0.4717	-0.8111 to -0.1322	0.0093
4D	PSR	Mycl vs. Mycn	-0.5038	-0.8432 to -0.1643	0.0061
4F	Myh7	GFP vs. Myc	-0.05025	-2.846 to 2.745	>0.9999
4F	Myh7	GFP vs. Mycl	0.2727	-2.523 to 3.068	0.9921
4F	Myh7	GFP vs. Mycn	-5.055	-7.851 to -2.260	0.0005
4F	Myh7	Myc vs. Mycl	0.3229	-2.472 to 3.118	0.9871
4F	Myh7	Myc vs. Mycn	-5.005	-7.800 to -2.210	0.0005
4F	Myh7	Mycl vs. Mycn	-5.328	-8.123 to -2.533	0.0003

Figure	Analysis	Comparison	Mean difference	95% CI	Adjusted p-Value
4F	Nppa	GFP vs. Mycl	0.326	-1.606 to 2.258	0.961
4F	Nppa	GFP vs. Mycn	-3.156	-5.205 to -1.106	0.0024
4F	Nppa	Myc vs. Mycl	1	-0.9322 to 2.932	0.4663
4F	Nppa	Myc vs. Mycn	-2.482	-4.531 to -0.4321	0.0155
4F	Nppa	Mycl vs. Mycn	-3.482	-5.531 to -1.432	0.001
4G	EF	GFP: pre vs. 4wk	-0.48	-9.887 to 8.927	>0.9999
4G	EF	GFP: pre vs. 8wk	0.2	-9.207 to 9.607	>0.9999
4G	EF	GFP: 4wk vs. 8wk	0.68	-8.727 to 10.09	>0.9999
4G	EF	Myc: pre vs. 4wk	-3.18	-12.59 to 6.227	0.9761
4G	EF	Myc: pre vs. 8wk	-5.67	-15.08 to 3.737	0.6026
4G	EF	Myc: 4wk vs. 8wk	-2.49	-11.90 to 6.917	0.9951
4G	EF	Mycn: pre vs. 4wk	-0.02727	-8.996 to 8.942	>0.9999
4G	EF	Mycn: pre vs. 8wk	-2.182	-11.15 to 6.787	0.9973
4G	EF	Mycn: 4wk vs. 8wk	-2.155	-11.12 to 6.814	0.9975
5E	Infarction	Mycn vs. GFP	4.15	0.06412 to 8.236	0.0471

Figure	Analysis	Comparison	Mean difference	95% CI	Adjusted p-Value
5F	EF	GFP-S-pre vs. GFP-S-4w	-4.333	-27.66 to 18.99	>0.9999
5F	EF	GFP-S-pre vs. GFP-M-pre	-2.758	-24.58 to 19.06	>0.9999
5F	EF	GFP-S-pre vs. GFP-M-2w	9.317	-12.50 to 31.13	0.9388
5F	EF	GFP-S-pre vs. GFP-M-4w	18.84	-2.976 to 40.66	0.1489
5F	EF	GFP-S-pre vs. Mycn-S-pre	-6.933	-30.26 to 16.39	0.9961
5F	EF	GFP-S-pre vs. Mycn-S-2w	6.233	-17.09 to 29.56	0.9984
5F	EF	GFP-S-pre vs. Mycn-S-4w	4.4	-18.92 to 27.72	>0.9999
5F	EF	GFP-S-pre vs. Mycn-M-pre	-2.458	-21.80 to 16.88	>0.9999
5F	EF	GFP-S-pre vs. Mycn-M-2w	6.429	-12.91 to 25.77	0.9901
5F	EF	GFP-S-pre vs. Mycn-M-4w	3.242	-16.10 to 22.58	>0.9999
5F	EF	GFP-S-2w vs. GFP-S-4w	-5.933	-29.26 to 17.39	0.999
5F	EF	GFP-S-2w vs. GFP-M-pre	-4.358	-26.18 to 17.46	0.9999
5F	EF	GFP-S-2w vs. GFP-M-2w	7.717	-14.10 to 29.53	0.9839
5F	EF	GFP-S-2w vs. GFP-M-4w	17.24	-4.576 to 39.06	0.2463
5F	EF	GFP-S-2w vs. Mycn-S-pre	-8.533	-31.86 to 14.79	0.9792
5F	EF	GFP-S-2w vs. Mycn-S-2w	4.633	-18.69 to 27.96	>0.9999
5F	EF	GFP-S-2w vs. Mycn-S-4w	2.8	-20.52 to 26.12	>0.9999
5F	EF	GFP-S-2w vs. Mycn-M-pre	-4.058	-23.40 to 15.28	0.9998
5F	EF	GFP-S-2w vs. Mycn-M-2w	4.829	-14.51 to 24.17	0.9992
5F	EF	GFP-S-2w vs. Mycn-M-4w	1.642	-17.70 to 20.98	>0.9999
5F	EF	GFP-S-4w vs. GFP-M-pre	1.575	-20.24 to 23.39	>0.9999
5F	EF	GFP-S-4w vs. GFP-M-2w	13.65	-8.168 to 35.47	0.5832
5F	EF	GFP-S-4w vs. GFP-M-4w	23.18	1.357 to 44.99	0.0288
5F	EF	GFP-S-4w vs. Mycn-S-pre	-2.6	-25.92 to 20.72	>0.9999
5F	EF	GFP-S-4w vs. Mycn-S-2w	10.57	-12.76 to 33.89	0.9114
5F	EF	GFP-S-4w vs. Mycn-S-4w	8.733	-14.59 to 32.06	0.9753
5F	EF	GFP-S-4w vs. Mycn-M-pre	1.875	-17.46 to 21.21	>0.9999
5F	EF	GFP-S-4w vs. Mycn-M-2w	10.76	-8.577 to 30.10	0.7379
5F	EF	GFP-S-4w vs. Mycn-M-4w	7.575	-11.76 to 26.91	0.9659
5F	EF	GFP-M-pre vs. GFP-M-2w	12.08	-8.124 to 32.27	0.6472
5F	EF	GFP-M-pre vs. GFP-M-4w	21.6	1.401 to 41.80	0.027
5F	EF	GFP-M-pre vs. Mycn-S-pre	-4.175	-25.99 to 17.64	>0.9999
5F	EF	GFP-M-pre vs. Mycn-S-2w	8.992	-12.83 to 30.81	0.9516
5F	EF	GFP-M-pre vs. Mycn-S-4w	7.158	-14.66 to 28.98	0.9911
5F	EF	GFP-M-pre vs. Mycn-M-pre	0.3	-17.19 to 17.79	>0.9999
5F	EF	GFP-M-pre vs. Mycn-M-2w	9.188	-8.306 to 26.68	0.8002
5F	EF	GFP-M-pre vs. Mycn-M-4w	6	-11.49 to 23.49	0.9873
5F	EF	GFP-M-2w vs. GFP-M-4w	9.525	-10.67 to 29.72	0.8877
5F	EF	GFP-M-2w vs. Mycn-S-pre	-16.25	-38.07 to 5.568	0.3251
5F	EF	GFP-M-2w vs. Mycn-S-2w	-3.083	-24.90 to 18.73	>0.9999
5F	EF	GFP-M-2w vs. Mycn-S-4w	-4.917	-26.73 to 16.90	0.9997
5F	EF	GFP-M-2w vs. Mycn-M-pre	-11.78	-29.27 to 5.718	0.4743
5F	EF	GFP-M-2w vs. Mycn-M-2w	-2.888	-20.38 to 14.61	>0.9999
5F	EF	GFP-M-2w vs. Mycn-M-4w	-6.075	-23.57 to 11.42	0.986
5F	EF	GFP-M-4w vs. Mycn-S-pre	-25.78	-47.59 to -3.957	0.0093
5F	EF	GFP-M-4w vs. Mycn-S-2w	-12.61	-34.43 to 9.209	0.6919
5F	EF	GFP-M-4w vs. Mycn-S-4w	-14.44	-36.26 to 7.376	0.4996
5F	EF	GFP-M-4w vs. Mycn-M-pre	-21.3	-38.79 to -3.807	0.0065
5F	EF	GFP-M-4w vs. Mycn-M-2w	-12.41	-29.91 to 5.081	0.3952
5F	EF	GFP-M-4w vs. Mycn-M-4w	-15.6	-33.09 to 1.893	0.1206
5F	EF	Mycn-S-pre vs. Mycn-S-2w	13.17	-10.16 to 36.49	0.721
5F	EF	Mycn-S-pre vs. Mycn-S-4w	11.33	-11.99 to 34.66	0.867

Figure	Analysis	Comparison	Mean difference	95% CI	Adjusted p-Value
5F	EF	Mycn-S-pre vs. Mycn-M-2w	13.36	-5.977 to 32.70	0.4349
5F	EF	Mycn-S-pre vs. Mycn-M-4w	10.18	-9.164 to 29.51	0.7985
5F	EF	Mycn-S-2w vs. Mycn-S-4w	-1.833	-25.16 to 21.49	>0.9999
5F	EF	Mycn-S-2w vs. Mycn-M-pre	-8.692	-28.03 to 10.65	0.9156
5F	EF	Mycn-S-2w vs. Mycn-M-2w	0.1958	-19.14 to 19.54	>0.9999
5F	EF	Mycn-S-2w vs. Mycn-M-4w	-2.992	-22.33 to 16.35	>0.9999
5F	EF	Mycn-S-4w vs. Mycn-M-pre	-6.858	-26.20 to 12.48	0.9836
5F	EF	Mycn-S-4w vs. Mycn-M-2w	2.029	-17.31 to 21.37	>0.9999
5F	EF	Mycn-S-4w vs. Mycn-M-4w	-1.158	-20.50 to 18.18	>0.9999
5F	EF	Mycn-M-pre vs. Mycn-M-2w	8.888	-5.396 to 23.17	0.591
5F	EF	Mycn-M-pre vs. Mycn-M-4w	5.7	-8.583 to 19.98	0.9612
5F	EF	Mycn-M-2w vs. Mycn-M-4w	-3.188	-17.47 to 11.10	0.9997

Table S3. GO enrichment

GOBP	GO.term	Ref.number	Gene.number			Fold.Enrichment			FDR		
			Mycn	Mycn	Mycl	Mycn	Mycn	Mycl	Mycn	Mycn	Mycl
**cell division**	GO:0051301	506	53	32	10	7.4	16.4	18.9	2.5E-25	2.3E-26	6.3E-07
**regulation of cell cycle**	GO:0051726	1067	66	37	9	4.4	9.0	8.1	2.2E-20	1.7E-22	4.6E-04
**nuclear division**	GO:0000280	317	46	24	7	10.2	19.6	21.1	7.2E-27	1.3E-20	5.5E-05
**chromosome segregation**	GO:0007059	282	43	17	5	10.7	15.6	17.0	8.8E-26	1.4E-12	4.1E-03
**mitotic cell cycle process**	GO:1903047	503	61	37	9	8.5	19.0	17.1	2.3E-32	6.0E-33	6.0E-06
**cell cycle**	GO:0007049	1248	89	44	12	5.0	9.1	9.2	2.8E-32	3.4E-28	6.9E-06
**non-membrane-bounded organelle assembly**	GO:0140694	306	22	15	7	5.1	12.7	21.9	3.2E-07	7.7E-10	5.0E-05
**microtubule-based process**	GO:0007017	812	41	22	NA	3.6	7.0	NA	1.7E-09	2.9E-10	NA
**regulation of chromosome segregation**	GO:0051983	126	23	10	6	12.8	20.5	45.5	1.3E-14	4.8E-08	1.2E-05
**establishment of chromosome localization**	GO:0051303	72	11	6	NA	10.7	21.6	NA	3.6E-06	1.0E-04	NA
**chromosome localization**	GO:0050000	77	11	6	NA	10.0	20.2	NA	6.5E-06	1.4E-04	NA
**cellular response to DNA damage stimulus**	GO:0006974	709	31	14	NA	3.1	5.1	NA	9.8E-06	1.1E-04	NA
**reproduction**	GO:0000003	1453	45	16	NA	2.2	2.9	NA	1.9E-04	0.0154	NA
**DNA replication**	GO:0006260	183	14	6	NA	5.4	8.5	NA	9.2E-05	0.0112	NA
**biological process involved in interspecies interaction between organisms**	GO:0044419	1646	47	NA	NA	2.0	NA	NA	6.1E-04	NA	NA
**cell adhesion**	GO:0007155	887	31	NA	NA	2.5	NA	NA	6.8E-04	NA	NA
**antigen processing and presentation of peptide antigen**	GO:0048002	81	7	NA	NA	6.1	NA	NA	0.011	NA	NA
**immune system process**	GO:0002376	2589	60	NA	NA	1.6	NA	NA	0.00941	NA	NA
**collagen metabolic process**	GO:0032963	69	9	NA	NA	9.2	NA	NA	1.6E-04	NA	NA
**establishment or maintenance of cell polarity**	GO:0007163	213	11	NA	NA	3.6	NA	NA	0.0148	NA	NA
**apoptotic process**	GO:0006915	983	27	NA	NA	1.9	NA	NA	0.0446	NA	NA
**cell population proliferation**	GO:0008283	858	27	NA	NA	2.2	NA	NA	0.00895	NA	NA

NA indicates that the corresponding GO term was not significantly enriched (FDR ≥ 0.05) in that condition.

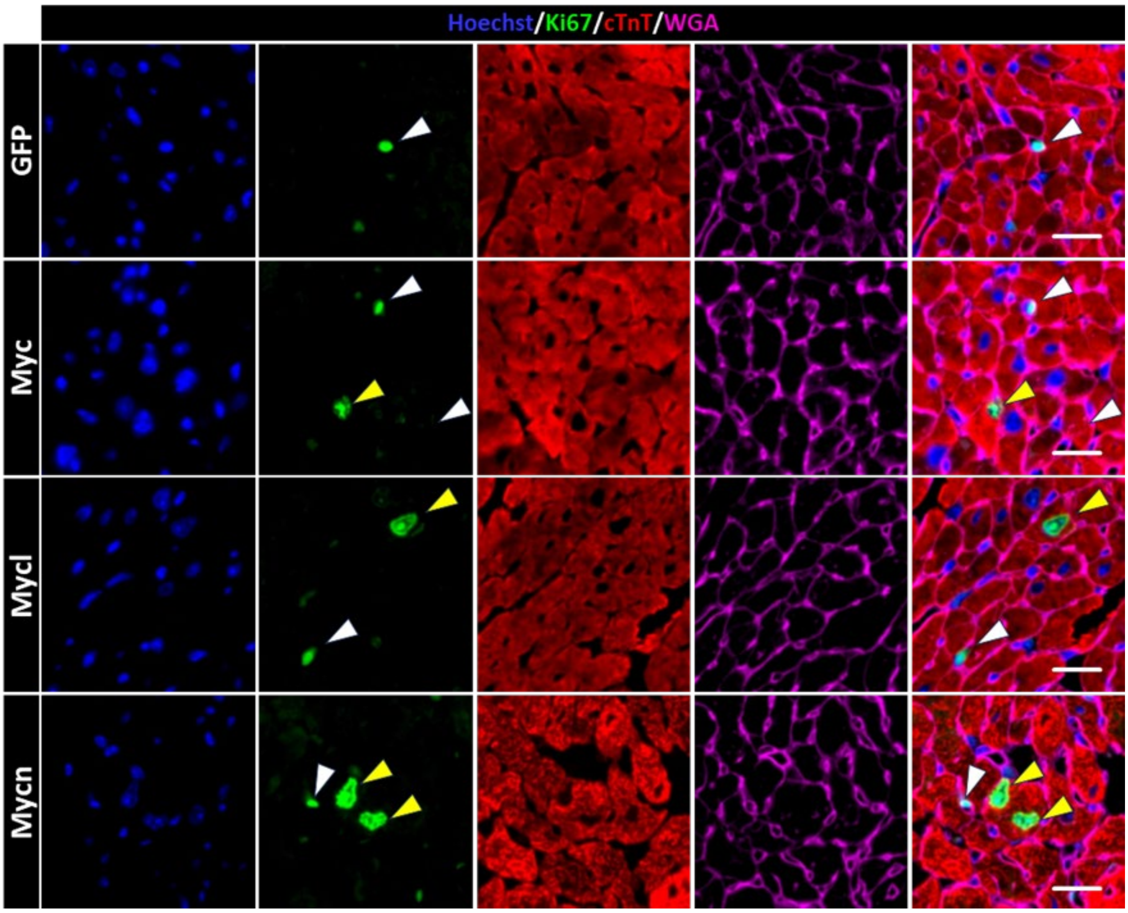

Figure S1. Identification of Ki67-positive cardiomyocytes: To assess whether Myc family gene expression induces cardiomyocyte cell-cycle activation, Ki67 immunofluorescence staining was performed. Cardiomyocyte nuclei were identified by co-immunostaining for cardiac troponin T (cTnT) and wheat germ agglutinin (WGA). Ki67-positive cardiomyocytes were observed in all Myc family groups, whereas only Ki67-positive non-cardiomyocytes were detected in the GFP group. Ki67 is shown in green, cTnT in red, Hoechst in blue, and WGA in magenta. Yellow arrowheads indicate Ki67-positive cardiomyocyte nuclei and white arrowheads indicate Ki67-positive non-cardiomyocyte nuclei. Scale bar: 20 µm.

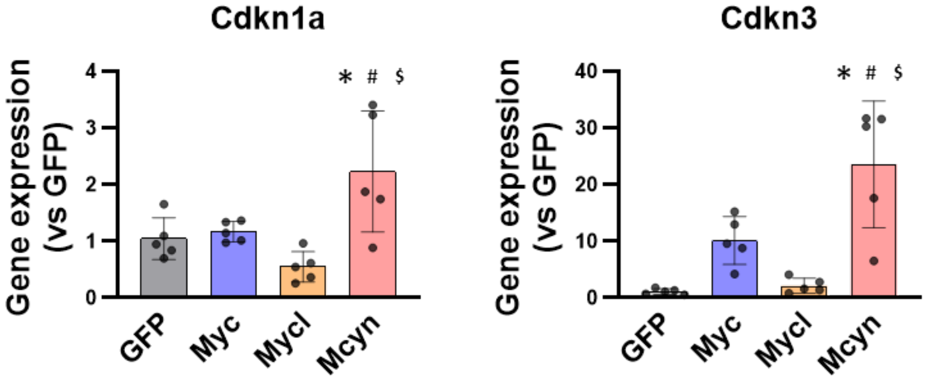

Figure S2. Significant upregulation of cell cycle inhibitors: To identify potential cell cycle–restrictive factors in Mycn-expressing cardiomyocytes, RNA-seq data were reanalyzed, revealing significant upregulation of Cdkn1a (p21) and Cdkn3, both cyclin-dependent kinase inhibitors. These findings were validated by RT-qPCR. Each dot represents an individual biological replicate. Statistical significance was assessed using one-way ANOVA followed by Tukey's post hoc test. $^{*}p<.05$ vs. GFP, $^{\#}p<.05$ vs. Myc, $^{\$}p<.05$ vs. Mycl.

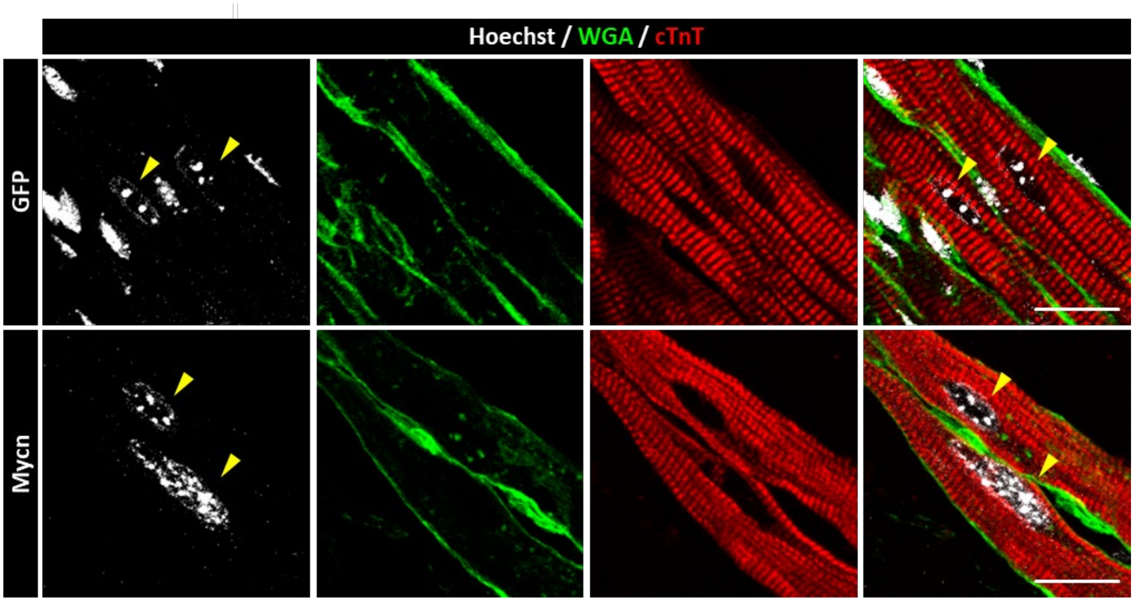

Figure S3. Altered heterochromatin morphology in Mycn-expressing cardiomyocytes: Morphological changes in cardiomyocyte nuclei were assessed by DNA and immunofluorescence staining. DNA was labeled with Hoechst 33342 to visualize heterochromatin, and cardiomyocyte nuclei were identified by co-immunostaining for cardiac troponin T (cTnT) and wheat germ agglutinin (WGA). GFP-expressing control cardiomyocytes exhibited typical oval nuclei with a few large heterochromatic foci, whereas Mycn-expressing cardiomyocytes displayed enlarged nuclei with a speckled pattern and increased heterochromatic foci. Hoechst is shown in blue, WGA in green, and cTnT in red; Yellow arrowheads indicate cardiomyocyte nuclei. Scale bar: 20 µm.

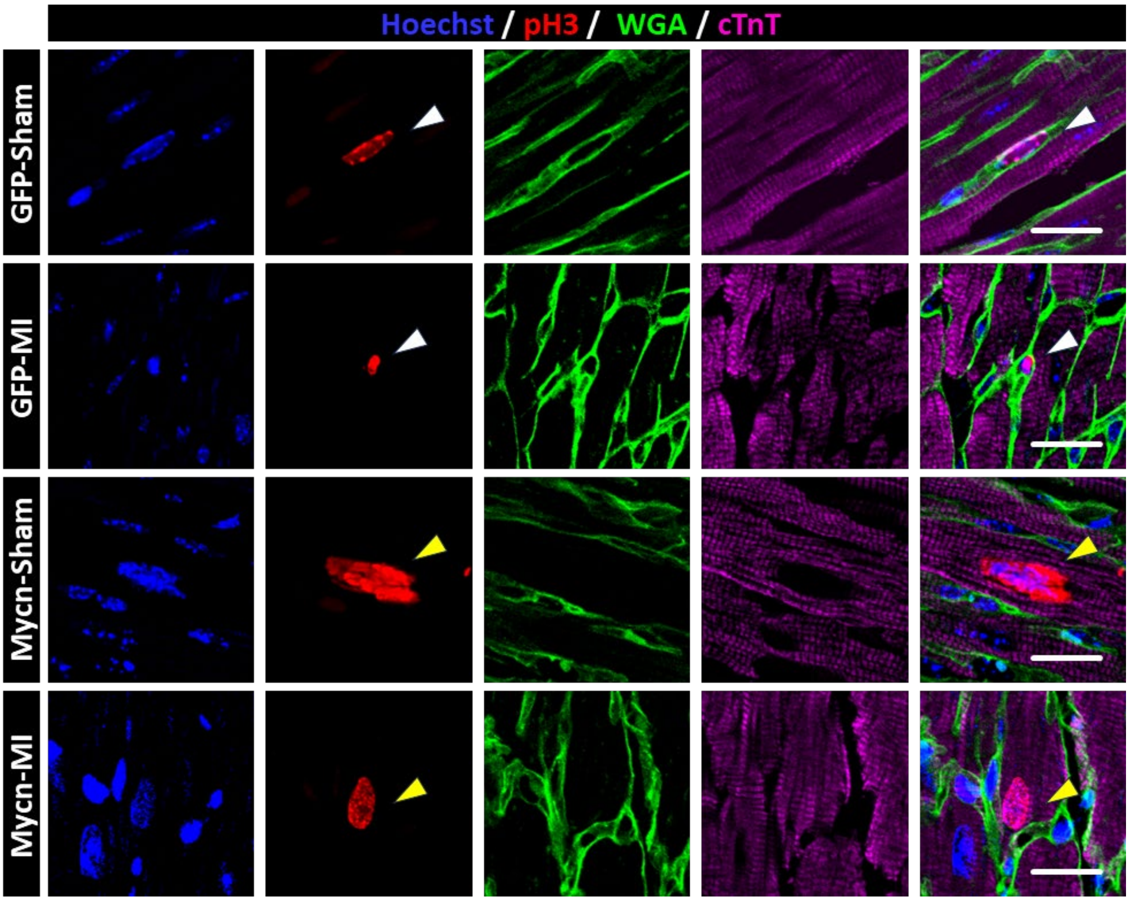

Figure S4. Mycn-induced cell cycle activation in MI condition: To evaluate whether Mycn activates the cardiomyocyte cell cycle following myocardial infarction (MI), phospho-H3 (pH3) immunofluorescence staining was performed. Cardiomyocyte nuclei were identified by co-immunostaining for cardiac troponin T (cTnT) and wheat germ agglutinin (WGA). Consistent with baseline findings, pH3-positive cardiomyocytes were observed in the Mycn group in both sham and MI hearts, whereas none were detected in the GFP group under either condition. pH3 is shown in red, Hoechst in blue, cTnT in magenta, and WGA in green. Yellow arrowheads indicate pH3-positive cardiomyocyte nuclei; white arrowheads indicate pH3-positive non-cardiomyocyte nuclei. Scale bar: 20 µm.

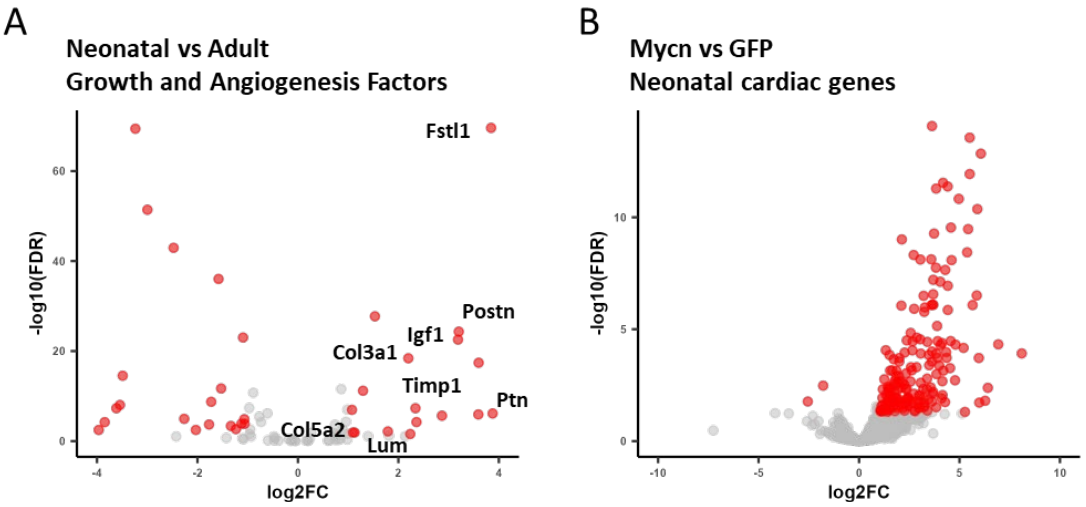

Figure S5. Mycn induces a neonatal-like transcriptional program in adult cardiomyocytes:

A. ***Mycn*-induced growth and angiogenic factors are highly expressed in neonatal cardiomyocytes:** To assess the developmental expression patterns of Mycn-induced growth and angiogenic factors, a previously published RNA-seq dataset (NCBI Accession: SRP033386) comparing neonatal (P1) and adult cardiomyocytes was reanalyzed using the same pipeline described in the Methods section. Genes associated with the Gene Ontology term "growth factor activity" (GO:0008083) or included in a curated angiogenesis gene set were visualized in a volcano plot. Mycn-induced growth and angiogenic factors were specifically labeled, revealing that a substantial number of these growth and angiogenesis-related factors are also highly expressed in neonatal cardiomyocytes. Genes with a false discovery rate (FDR) < 0.05 and a fold change (FC) ≥ 2 (neonatal cardiomyocytes vs adult cardiomyocytes) are shown in red.

B. **Upregulation of neonatal genes in *Mycn*-expressing cardiomyocytes:** Using the same RNA-seq dataset as in Supplemental Figure 1A, neonatal cardiac genes were defined as those significantly upregulated in P1 cardiomyocytes relative to adult cardiomyocytes (false discovery rate (FDR) < 0.05 and absolute fold change (FC) ≥ 2), and compiled into a neonatal gene list. Differential expression analysis was then performed between *Mycn*- and *GFP*-expressing cardiomyocytes to assess the expression of these neonatal genes. The results were visualized as a volcano plot. Of the 367 genes upregulated in the Mycn group, 188 genes (51%) overlapped with the neonatal gene list, a phenotypic shift toward a neonatal-like cardiomyocyte state.

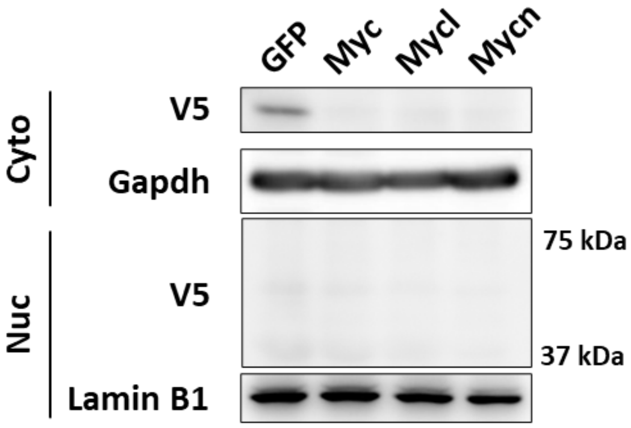

Figure S6. Low protein abundance of Myc family transgenes: To assess Myc family transgene expression at the protein level, Western blotting was performed on purified cardiomyocytes using an anti-V5 antibody. GFP was detected in the cytoplasmic fraction (Cyto), whereas Myc, Mycl, and Mycn proteins were below the detection threshold in the nuclear-enriched fraction (Nuc). The expected molecular weights of Myc-V5, Mycl-V5, and Mycn-V5 are 53.5 kDa, 45.4 kDa, and 54.2 kDa, respectively.

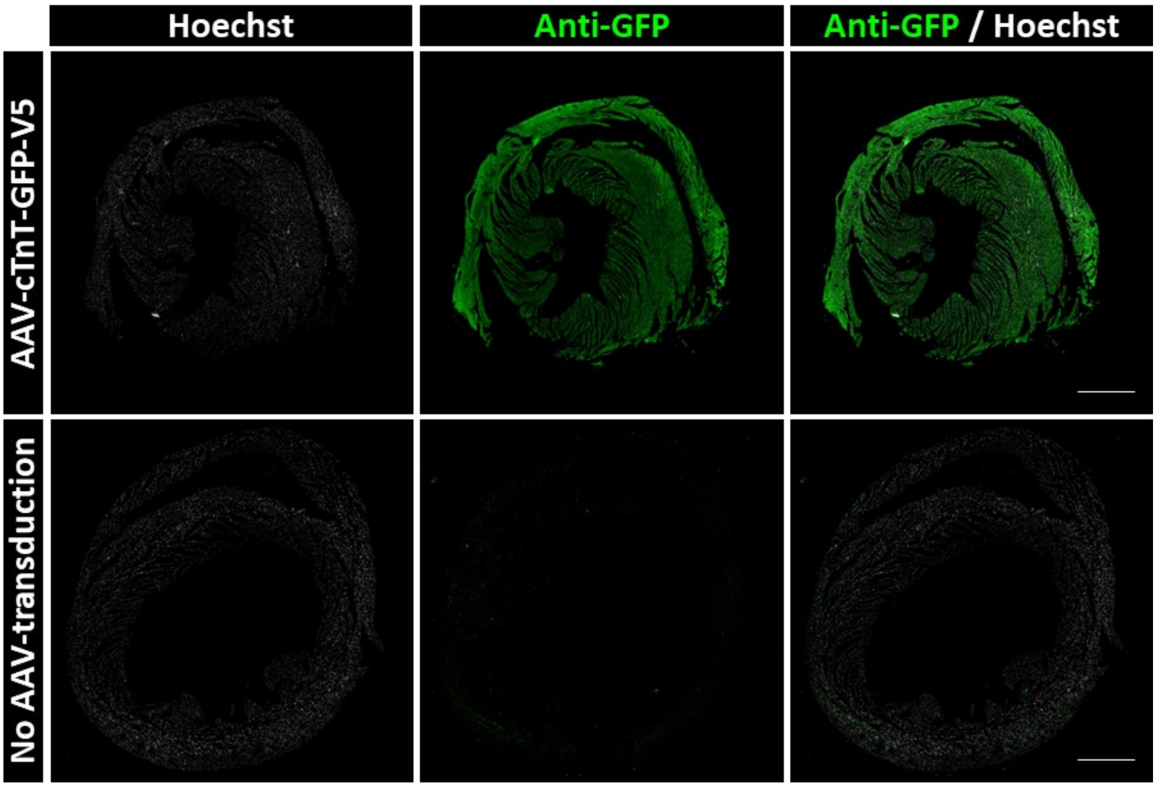

Figure S7. Confirming AAV9-mediated gene transduction efficiency: To evaluate AAV9-mediated gene transduction efficiency, immunofluorescence staining with an anti-GFP antibody was performed on heart sections from GFP groups at 2 weeks. GFP expression was uniformly distributed throughout the myocardium, whereas no GFP signal was detected in non–AAV-transduced control hearts. GFP is shown in Green, and Hoechst in white. Scale bar: 1 mm.
