# Supplementary material for: Mycn Reactivates the Cell Cycle in Adult Cardiomyocytes and Promotes Cardioprotection in Myocardial Infarction
Source: J Am Heart Assoc. 2026 Apr 9;15(8):e046146. doi: 10.1161/JAHA.125.046146 (PMC13279142; doi:10.1161/JAHA.125.046146)

Full unedited blots

Full unedited blot for Supplemental Figure 6

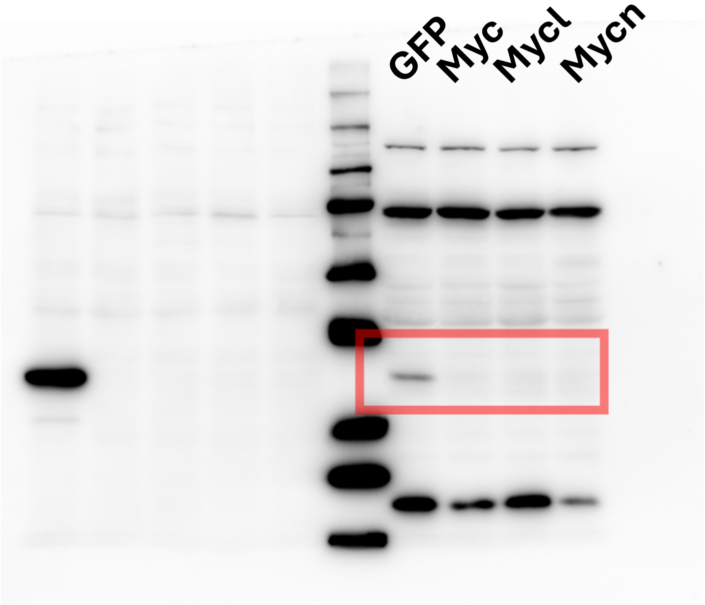

Full unedited blot for Supplemental Figure 6

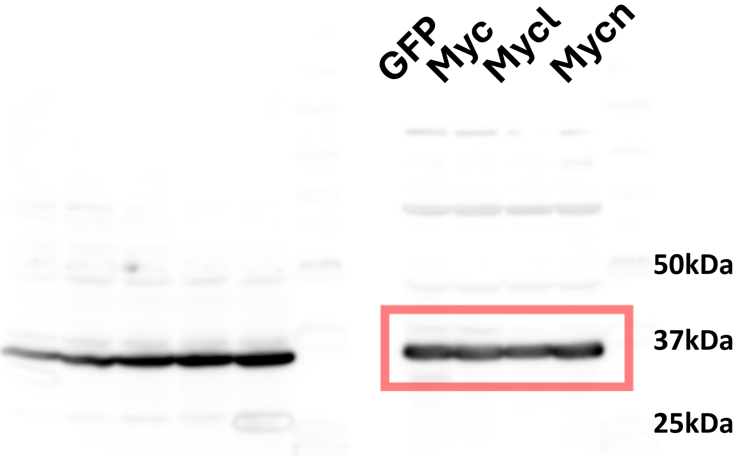

Full unedited blot for Supplemental Figure 6

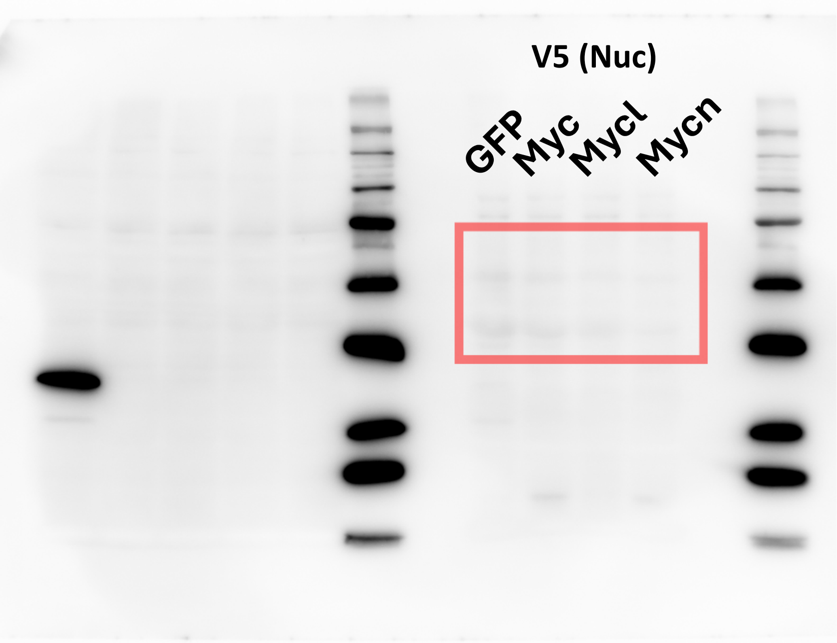

Full unedited blot for Supplemental Figure 6

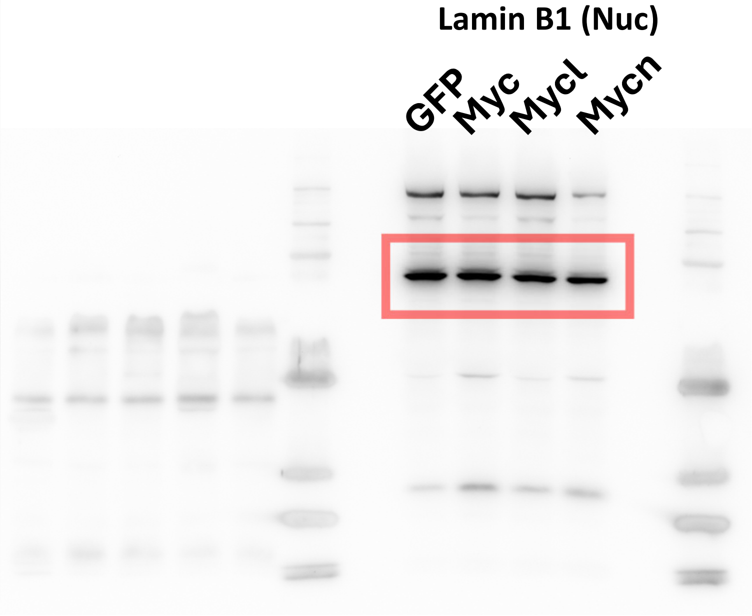

Supplement: Supplementary file 2 — Unedited Blots [file JAH3-15-e046146-s001.pdf]
